# Kinetic regulation of multi-ligand binding proteins

**DOI:** 10.1186/s12918-016-0277-0

**Published:** 2016-04-18

**Authors:** Diana V. Salakhieva, Ildar I. Sadreev, Michael Z. Q. Chen, Yoshinori Umezawa, Aleksandr I. Evstifeev, Gavin I. Welsh, Nikolay V. Kotov

**Affiliations:** Kazan (Volga Region) Federal University, 18 Kremlyovskaya St., 420008 Kazan, Russia; Centre for Systems, Dynamics and Control, College of Engineering, Mathematics and Physical Sciences, University of Exeter, Harrison Building, North Park Road, Exeter, EX4 4QF UK; Department of Mechanical Engineering, The University of Hong Kong, Pokfulam Road, Hong Kong, China; Department of Dermatology, The Jikei University School of Medicine, 3-25-8 Nishishimbashi, Minato-ku, Tokyo, 105-8461 Japan; Biophysics & Bionics Lab, Institute of Physics, Kazan Federal University, Kazan, 420008 Russia; Academic Renal Unit, School of Clinical Sciences, University of Bristol, Dorothy Hodgkin Building, Whitson Street, Bristol, BS1 3NY UK

**Keywords:** Ligand-receptor binding, Transient kinetics, Calcium, Calmodulin

## Abstract

**Background:**

Second messengers, such as calcium, regulate the activity of multisite binding proteins in a concentration-dependent manner. For example, calcium binding has been shown to induce conformational transitions in the calcium-dependent protein calmodulin, under steady state conditions. However, intracellular concentrations of these second messengers are often subject to rapid change. The mechanisms underlying dynamic ligand-dependent regulation of multisite proteins require further elucidation.

**Results:**

In this study, a computational analysis of multisite protein kinetics in response to rapid changes in ligand concentrations is presented. Two major physiological scenarios are investigated: i) Ligand concentration is abundant and the ligand-multisite protein binding does not affect free ligand concentration, ii) Ligand concentration is of the same order of magnitude as the interacting multisite protein concentration and does not change. Therefore, buffering effects significantly influence the amounts of free ligands. For each of these scenarios the influence of the number of binding sites, the temporal effects on intermediate apo- and fully saturated conformations and the multisite regulatory effects on target proteins are investigated.

**Conclusions:**

The developed models allow for a novel and accurate interpretation of concentration and pressure jump-dependent kinetic experiments. The presented model makes predictions for the temporal distribution of multisite protein conformations in complex with variable numbers of ligands. Furthermore, it derives the characteristic time and the dynamics for the kinetic responses elicited by a ligand concentration change as a function of ligand concentration and the number of ligand binding sites. Effector proteins regulated by multisite ligand binding are shown to depend on ligand concentration in a highly nonlinear fashion.

## Background

A wide variety of intracellular events are initiated via temporal change of ligand concentrations. One of the most important ligands in many cells is calcium (Ca^2+^). Calcium interacts with and regulates the activities of a large number of calcium-binding proteins as well as numerous effectors. The number of functional calcium binding sites within these proteins can range from one or two to ten or more [1–3]. The most common number of calcium binding sites is four: as observed in the most ubiquitous protein, calmodulin as well as troponin and other EF-hand containing proteins [4, 5]. Temporal elevation of intracellular free Ca^2+^ is the key regulatory factor of the Ca^2+^-dependent protein activity [6–10]. The characteristics of the induced signal are not fully understood. It remains to be determined how a single ligand is able to govern numerous intracellular properties.

Whilst the multisite ligand binding is not limited to Ca^2+^ signaling, Ca^2+^ is probably the most versatile ion, regulating the largest number of cellular events. Several Ca^2+^-binding proteins can be considered as examples of multisite ligand protein interactions. Structural biology investigations of calcium binding proteins in complexes with target protein peptides have suggested that the specificity in Ca^2+^-CaM binding protein-dependent target activation arises from the diversity of interaction interfaces between the Ca^2+^-regulated protein and its target proteins [5, 11–21]. The most ubiquitous protein, calmodulin (CaM), consists of two globular domains, each domain containing a pair of helix-loop-helix Ca^2+^-binding motifs called EF-hands [1, 3, 5, 16, 17]. In earlier studies the authors demonstrated that in addition to the diversity of CaM-target interfaces; the CaM selectivity emerges from its target specific Ca^2+^-affinity; the number of Ca^2+^ ions bound and the target specific cooperativity [22–24].

Another major factor that contributes to the selectivity of seemingly simultaneous regulation of several multisite Ca^2+^ binding proteins and Ca^2+^-mediated processes is the temporal alterations of Ca^2+^ [25–27]. The remarkable variety of Ca^2+^ signals in cells, ranging from infrequent spikes to sustained oscillations and plateaus, requires an understanding of how fast intracellular calcium changes regulate the kinetics of multiple multisite Ca^2+^ binding proteins. Therefore mathematical modeling of Ca^2+^ jump induced responses could prove to be invaluable in the interpretation of transient kinetic experiments.

Cooperative binding is a special case of molecular interactions where ligand binding to one site of a molecule depends on the ligand binding to the other sites. The first quantitative determination of the dynamic properties of cooperative binding was proposed by [28]. In this work the authors emphasized the significance of the cooperativity by studying the fast dynamics of Ca^2+^ binding to calretenin (CR), which has one independent and four cooperative binding sites. The investigation of cooperative effects of Ca^2+^ binding to CR was performed both experimentally and using mathematical modeling. The authors employed the simplified version of the Adair-Klotz model [29, 30] to describe the dynamics of the interactions involved in Ca^2+^ binding to CR. This approach was then extended to the binding of Ca^2+^ to CaM [31]. The models proposed in these studies [28, 31] demonstrated excellent fitting results to the experimental data, in comparison with the previously published models. However, the described approach is rather limited, as it describes fitting instead of providing a mechanistic description. An alternative methodology offered by [28, 31] is not directly applicable from the physical and chemical point of view because the Adair-Klotz model for sequential ligand binding was utilized [29, 30] whereas binding of Ca^2+^ to EF-hand proteins [32–35] is non-sequential [22]. Given the importance of studying fast Ca^2+^ binding kinetics and the lack of understanding of the underlying mechanisms, we developed a detailed mathematical model for ligand binding to multisite proteins with both cooperative and independent binding sites.

Mathematical modelling of multisite protein kinetics in response to rapid ligand changes presented in this paper provide new insights into the mechanism of conformational kinetics of multisite proteins in complex with variable number of bound ligands for the two distinct physiological situations described:

i) When the ligand concentration significantly exceeds protein concentration,
ii) When the total amount of ligand is conserved and comparable with the protein concentration.

In the first case, the buffering effects are negligible whereas in the latter, the ligand-protein interactions have a significant impact on the amount of available ligand and the binding kinetics. In this work, the equations for the dependence of the characteristic time constants and the temporal distribution of individual conformations as a function of the ligand concentration, the number of binding sites and the binding affinities have been derived. The impact of the number of binding sites, temporal effects on conformations, and regulation by multisite proteins of their effector proteins have been investigated by employing the developed models. The analysis of the ligand-multisite protein mediated regulation of effector proteins suggests that significant degree of selectivity in regulation can be achieved by a single ligand by employing mechanisms described in this study.

## Results

### A new model for multisite protein ligand binding kinetics

The majority of studies of the activation of multisite proteins consider only the ligand concentration-dependent profiles. One of the interesting questions about these multisite proteins is how temporal alterations of ligand concentration contribute to their function. The shape of distribution of multisite proteins in complex with variable numbers of bound ligand is known or can be experimentally elucidated in many cases [4, 5, 16, 22–24, 36–38]. However, the role of temporal transitions caused by fast alteration of ligand concentration on multisite proteins and on multisite protein-regulated target proteins remains unclear. It is reasonable to assume that there can be at least two distinct mechanisms of fast ligand alteration-mediated effects exhibited in two distinct system scenarios: i) the ligand concentration is significantly greater than the multisite protein concentration, ii) the ligand is comparable with the multisite protein concentration. In the first scenario ligand binding to multisite protein leads to insignificant changes of free ligand, whereas in the second case, free ligand concentration can vary substantially when ligand molecules bind to the multisite proteins. This paper describes the development of the two models, which address these distinct physiological situations.

## The model for abundant ligand concentration

In this model we describe physiological situations where the ligand concentration significantly exceeds the multisite protein concentration. In a previous study [22, 24] the authors analyzed functions for the probability of an individual site being in the bound or non-bound state and a function giving the probability of a multisite protein being in a complex with different number of bound ligands (Eqs. (2) and (3) in Methods) [29, 39, 40]. To investigate the kinetics of the multisite protein ligand interactions the present study extends the previous model to consider the ligand concentration as a function of time (Eqs. (4) and (5) in Methods). The solutions for the individual sites to be in a particular state were obtained for those cases where ligands are subject to rapid changes between steady-states (Eqs. (6) and (7) in Methods). Due to the large number of sites involved, knowledge of the state probability distribution for individual binding sites allows accurate estimation of the dynamics of the total concentration of bound ligand in response to a jump in free ligand concentration (Eq. (11) in Methods).

In order to gain more insights into the distribution of the intermediate protein conformations (complexes with variable number of bound ions) we investigated the case of a multisite protein with identical binding sites (Eq. (12) in Methods). While this case is a relatively rare occurrence in living cells, it enables insight into the role that the number of binding sites plays in cellular signalling. There are several examples of protein families that have variable number of ligand binding sites either due to their structural properties or by them forming large tertiary complexes. For example members of the Ca^2+^ family of binding proteins can differ in the number of ligand binding sites [41, 42]. The most ubiquitous Ca^2+^-binding protein, calmodulin (CaM), contains four Ca^2+^ binding sites as does troponin (TnC) [18] and calcineurin phosphatase (CaN) [43]. However the number of functional Ca^2+^ binding sites can vary from two to ten as in the protease, calpain [1] or even more in other cases [2]. To investigate the role that the number of ligand binding sites plays in multisite kinetics, the ligand concentrations, at which the intermediate conformations reach their maximum values, (Eq. (13) in Methods) and the corresponding magnitudes for those conformations (Eq. (14) in Methods) were estimated.

**Fig. 1:**
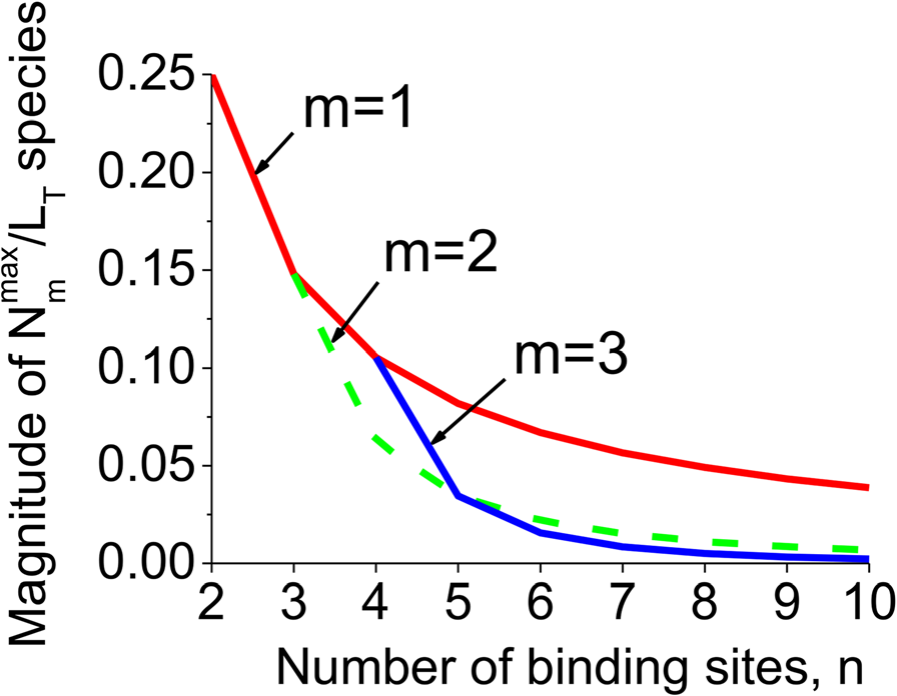
The effect of the number of binding sites on intermediate conformations. The maximum magnitude of protein conformations in complex with one, two and three ligand molecules are shown as a function of the total number of binding sites. The relative amount of ligand binding by conformations bound to a specific number of sites clearly diminishes as the number of sites grow

Figure 1 shows the maximum protein conformations in complex with one, two and three ligands as a function of the number of binding sites (Eq. (14) in Methods). The graph demonstrates that the magnitude of the ligand-multisite complexes decreases dramatically as the number of binding sites increases. The presented results suggest that the relative magnitude of individual intermediate conformations decreases as the number of binding sites increases. This in turn results in subtler regulatory effects of those proteins with larger number of ligand binding sites. For example, in CaM that has four binding sites for calcium [18], the presence of four sites leads to the increased multifunctionality of this protein due to the additional regulatory properties of intermediate conformations [24]. However, as an exception some forms of CaM have six binding sites [44, 45]. In this case, according to Fig. 1 the magnitude of intermediate conformations significantly decreases compared to the case of four binding sites, resulting in decreased regulatory properties of the protein. The presence of more than one binding site results in increased multifunctionality of the protein but at the same time leads the decrease of the regulatory effects. Thus, it seems that there is an “optimal” number of binding sites, which have been developed during the evolution, for instance four calcium binding sites in CaM.

**Fig. 2:**
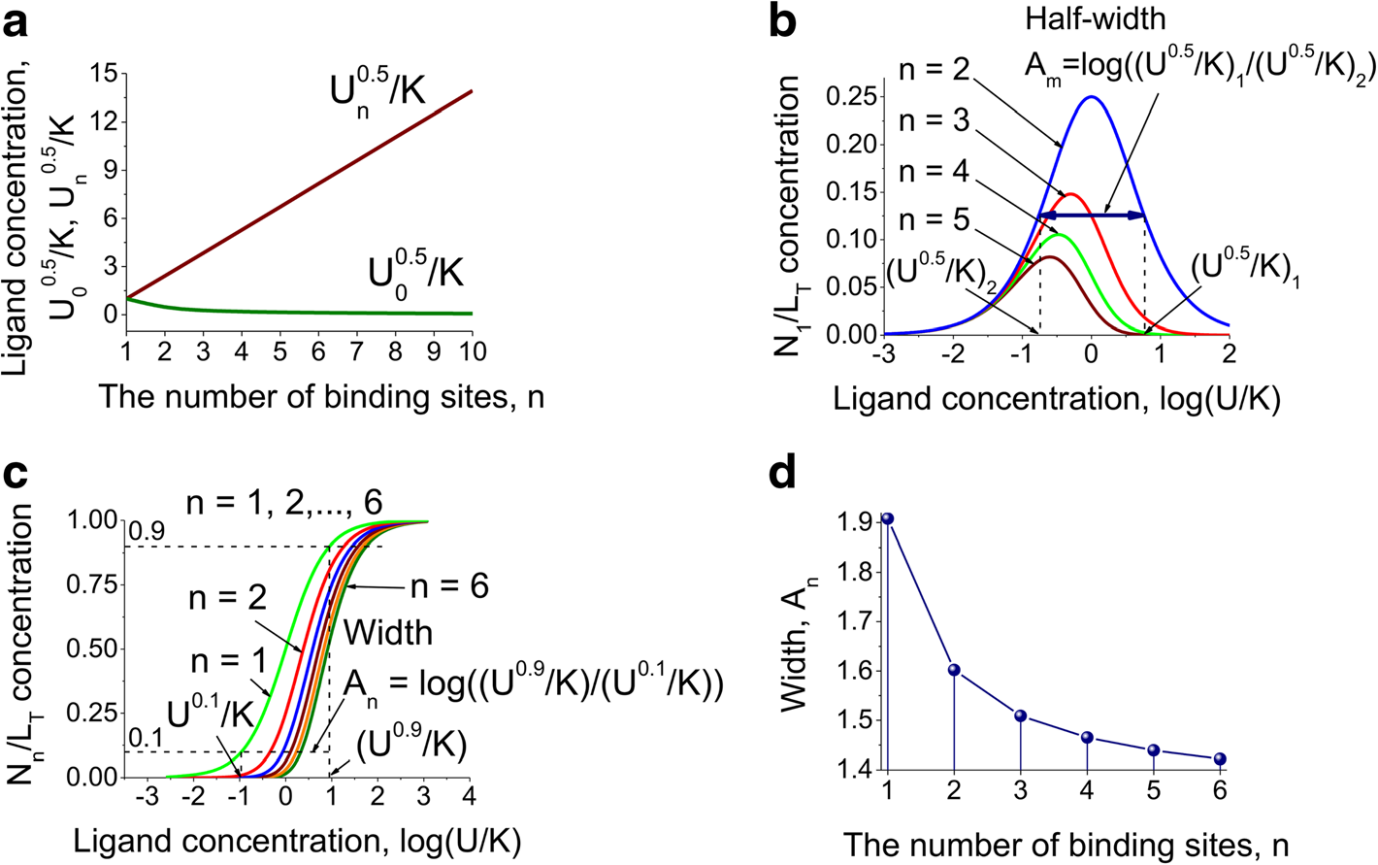
Model predictions for the half-maximal effective ligand concentrations as a function of the number of binding sites and ligand concentration. **a**. The dependence of the half-maximal effective ligand concentration, *U*_0_^0.5^/*K* and *U*_*n*_^0.5^/*K*, for the apo- and saturated multisite protein conformations respectively, on the number of binding sites. The effect of the increasing of the amount of binding sites is negligible for the fully bound conformation. **b**. Calculations for the half-width between the half-maximal effective ligand concentrations as a function of the ligand concentration for proteins with two, three, four and five binding sites. **c**. The difference between ligand concentrations for the saturated multisite protein conformations when the protein species equal to 90 % and 10 % of the total concentration as a function of the ligand concentration for the proteins with one to six binding sites. **d**. The difference between ligand concentrations for the saturated multisite protein conformations when the protein species equal to 90 % and 10 % of the total concentration as a function of the number of binding sites up to six

Next, the ligand concentrations for half maximum effective ligand concentrations, *U*_0_^0.5^ and *U*_*n*_^0.5^, for the apo- and saturated multisite protein conformations when the protein species equals 50 % of the total concentration were estimated (Eqs. (15) in Methods). This solution shows that the ligand concentration for the half maximal protein activity, known as *EC*_50_, would be equal to the equilibrium dissociation constant *K* (*EC*_50_ = *K*) for proteins with one binding site only (*n* = 1). Figure 2a shows the dependence of *U*_0_^0.5^/*K* and *U*_*n*_^0.5^/*K*, on the number of binding sites. The model predicts that there is a significant change in the required ligand concentration *U*_*n*_^0.5^/*K* for the fully bound conformation, while *U*_0_^0.5^/*K* does not change with time.

In the previous study the authors reported on the regulatory importance of the distribution of individual multisite protein conformations [22, 24]. Here calculations (Eqs. (15) in Methods) are presented for the half-width between the half-maximal effective ligand concentrations *U*^0.5^/*K* as a function of the ligand concentration for the intermediate conformations (one bound ligand) of the proteins with different number of binding sites (Fig. 2b). The observation of the half-width for the concentrations of intermediate conformations that have a bell-shaped dependence on ligand concentration, enables the range of physiologically plausible concentrations of ligand, where protein functions can be regulated by intermediate conformations to be obtained. For example, in Fig. 2b the half-width range of calcium concentrations is approximately from -1 to 1 on the logarithmic scale, which corresponds to 10^-7^M-10^-5^M due to the fact that the affinity of calcium binding sites in CaM is approximately 10^-6^M [46, 47]. Interestingly, the range 10^-7^M-10^-5^M corresponds to the physiological range of intracellular calcium concentrations in cardiac muscle cells [48]. Within this range of calcium concentrations, the switching between calcium channel opening and closure takes place [49].

The difference *A*_*n*_ between ligand concentrations *U*^0.9^/*K* and *U*^0.1^/*K* for the saturated multisite protein conformations, when the protein species are equal to 90 % and 10 % of the total concentration, as a function of the ligand concentration for proteins with different number of binding sites is shown in Fig. 2c and d respectively. The determination of *A*_*n*_ allows an understanding as to how an increase of the number of binding sites *n* affects the steepness of the dose-response curve (Fig. 2c). It can be seen from Fig. 2c and d that with an increase of *n*, the width *A*_*n*_ decreases while the steepness increases and shifts to the range of higher ligand concentrations *U*/*K*. The greater steepness of the dose-response curve caused by the presence of increasing number of binding sites (Fig. 2c) may result in the switch-like response and ultrasensitivity of the protein activation [50]. For instance, the steepness of CaM activation defines the threshold properties for the switching of erythrocyte aggregation and deformability from one steady-state to another [51].

Equation (17) for the total amount of bound ligand in the case of multisite protein with identical binding sites, can be used to estimate the amount of bound ligand when the ligand concentration is equal to the equilibrium dissociation constant (*U* = *K*). Our model predicts that the ligand concentration for the half maximal protein activation, *EC*_50_, is equal to the equilibrium dissociation constant *K* for any number of bound sites for a multisite protein with identical binding sites.

**Fig. 3:**
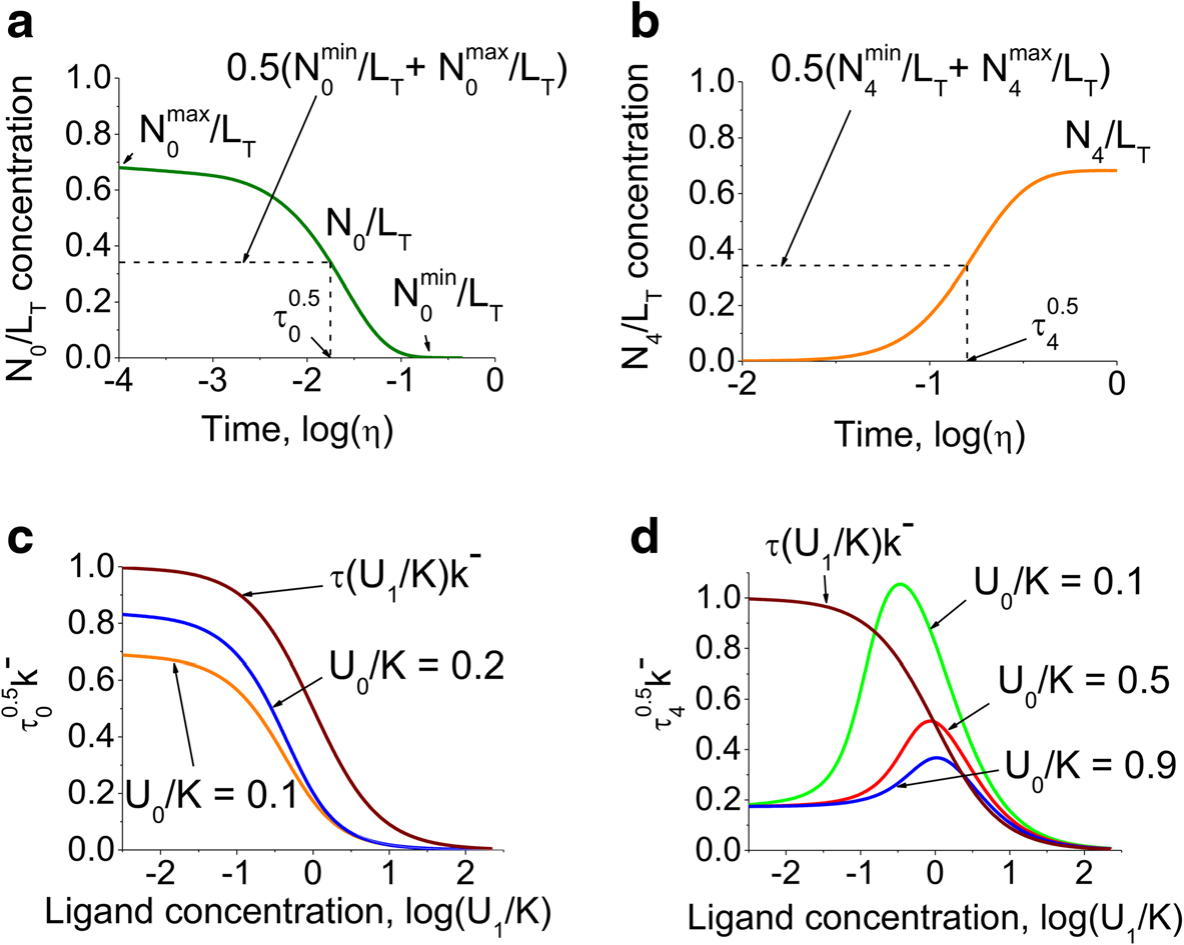
Temporal characteristics of the apo- and fully bound species in response to a ligand jump. The dynamics of the concentration of proteins was investigated for apo- (**a**) and fully bound (**b**) forms in response to the non-dimensional ligand concentration change from *U*_0_/*K* = 0.1 to *U*_1_/*K* = 10 as a function of non-dimensional time *η* = *t* ⋅ *k* ^−^. The dotted lines indicate the time, *τ*_0_^0.5^, required for the non-dimensional concentration of apo- conformation, *N*_0_/*L*_*T*_, to reach half of the fall in concentration and the period of time, *τ*_4_^0.5^, that takes for the fully saturated protein species, *N*_4_/*L*_*T*_, to gain half of the growth in concentration. These kinetic parameters then were subject to the investigation as a function of the initial (**c**) and final (**d**) ligand concentrations. The presented analysis clearly demonstrates the bell shaped dependence of *τ*_4_^0.5^ on the final ligand concentration

Figure 3 shows the calculations for the temporal characteristics of the apo- and fully bound species. Figure 3a and b show that the temporal shapes of the apo- and fully bound conformations (Eqs. (19) in Methods) in response to a ligand change are similar to the steady-state dependence of the same conformations on ligand concentration [24]. The kinetic parameters, *τ*_0_^0.5^ and *τ*_4_^0.5^ can be estimated as the time required to reach 50 % of the total concentration (Fig. 3a and b). The described kinetic parameters have been investigated as a function of the initial and final ligand concentrations (Fig. 3c, d and Eqs. (24) in Methods). Our analysis reveals a reduction of the time constant, *τ*_0_^0.5^, of the apo- conformation with the reduction of the initial ligand concentration and an increase of the final ligand concentration. However, the dependence of the characteristic time *τ*_4_^0.5^ (Fig. 3d) showed an unexpected bell shaped dependence on the final ligand concentration compared to the simpler monotonic dependence for *τ*_0_^0.5^ (Fig. 3c). The model predicts that there is an “optimal” ligand concentration for the saturation effect to take the longest time (Fig. 3d).

The bell shaped dependence of *τ*_4_^0.5^ for a protein with four binding sites, for example CaM [18], on final ligand concentration shown in Fig. 3d appears to be related to the presence of intermediate conformations with one, two and three bound sites. In a single site molecule (*n* = 1), for example crystalline bovine β-trypsin [52] that is characterized by the absence of intermediate forms, *τ*_4_^0.5^ depends on *U*_1_/*K* monotonically, i.e. there is no bell shaped dependence, as is evident from Eqs. (24). In CaM (*n* = 4), the activation of intermediate conformations takes extra time, which affects *τ*_4_^0.5^. This activation precedes the activation of the saturated form in time, and also as *U*_1_/*K* increases, i.e. the intermediate conformations are more prevalent for smaller values of *U*_1_/*K*, and the stationary distribution shifts towards the saturated form for larger *U*_1_/*K*. As a result, an increase of *U*_1_/*K* leads to an increase of the contribution of the kinetics of the intermediate complexes to the overall dynamics, and hence to an increase of *τ*_4_^0.5^. For larger *U*_1_/*K* the role of the intermediate conformations is less important and overall speed-up dominates, hence *τ*_4_^0.5^ decreases. Thus the model predicts that there is an intermediate ligand concentration, at which the kinetics of the fully saturated form, represented by *τ*_4_^0.5^, is the slowest.

### The affinities of binding sites differentially affect the kinetic responses of intermediate conformations

The proposed model has been employed to investigate the ligand jump-dependent kinetics of both saturated and non-saturated conformations. Initially, an idealized model of a multisite protein with identical binding sites was used to investigate the impact of ligand concentrations on the multisite protein kinetics. However, in living cells there are very few proteins (if any) that have identical ligand binding sites. Therefore the model was extended to examine the implications of variations in binding site affinities on the predicted concentration-response profiles.

**Fig. 4:**
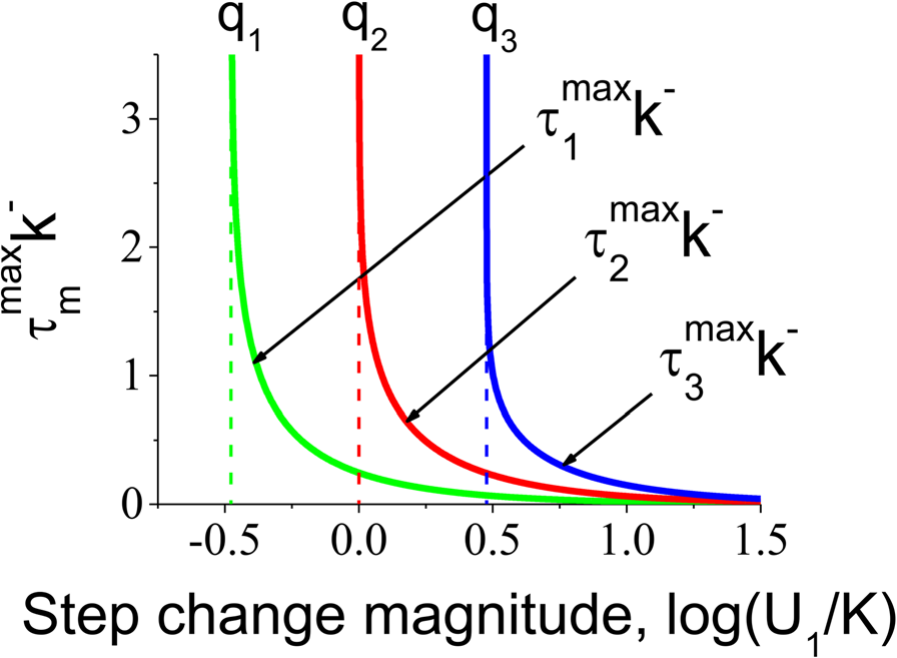
Characteristic time required for intermediate conformations to reach their maximum levels as a function of the step change magnitude. The analysis shows that the non-dimensional time (*η*_*m*_^*max*^ = *τ*_*m*_^*max*^ *k* ^−^, where *m* = 1, 2 and 3) required for reaching the maximum level of the intermediate species, is inversely proportional to the concentration of the applied ligand *U*_1_/*K*. This effect is due to the growing abundance of the free ligand concentration available for faster interaction with the multisite protein

Figure 4 shows the dependence of the time point *τ*_*m*_^*max*^*k*^−^ when the intermediate protein conformations reach the maximum as a function of magnitude of ligand jump (Eqs. (26) in Methods). It can be seen from Eqs. (26) that *τ*_1_^*max*^*k*^−^, *τ*_2_^*max*^*k*^−^ and *τ*_3_^*max*^*k*^−^ do not exist for ${U}_1/K<\frac{1}{3}$, *U*_1_/*K* < 1 and *U*_1_/*K* < 3, respectively. Under these special cases, where the ligand concentration *U*_1_/*K* is not sufficient for the concentrations of the intermediate conformations to reach their maximal values, these concentrations monotonously grow to their respective steady-state levels. According to Eq. (26), the values ${U}_1/K<\frac{1}{3},$*U*_1_/*K* < 1 and *U*_1_/*K* < 3 correspond to the three individual intermediate conformations with one, two and three bound sites respectively.

**Fig. 5:**
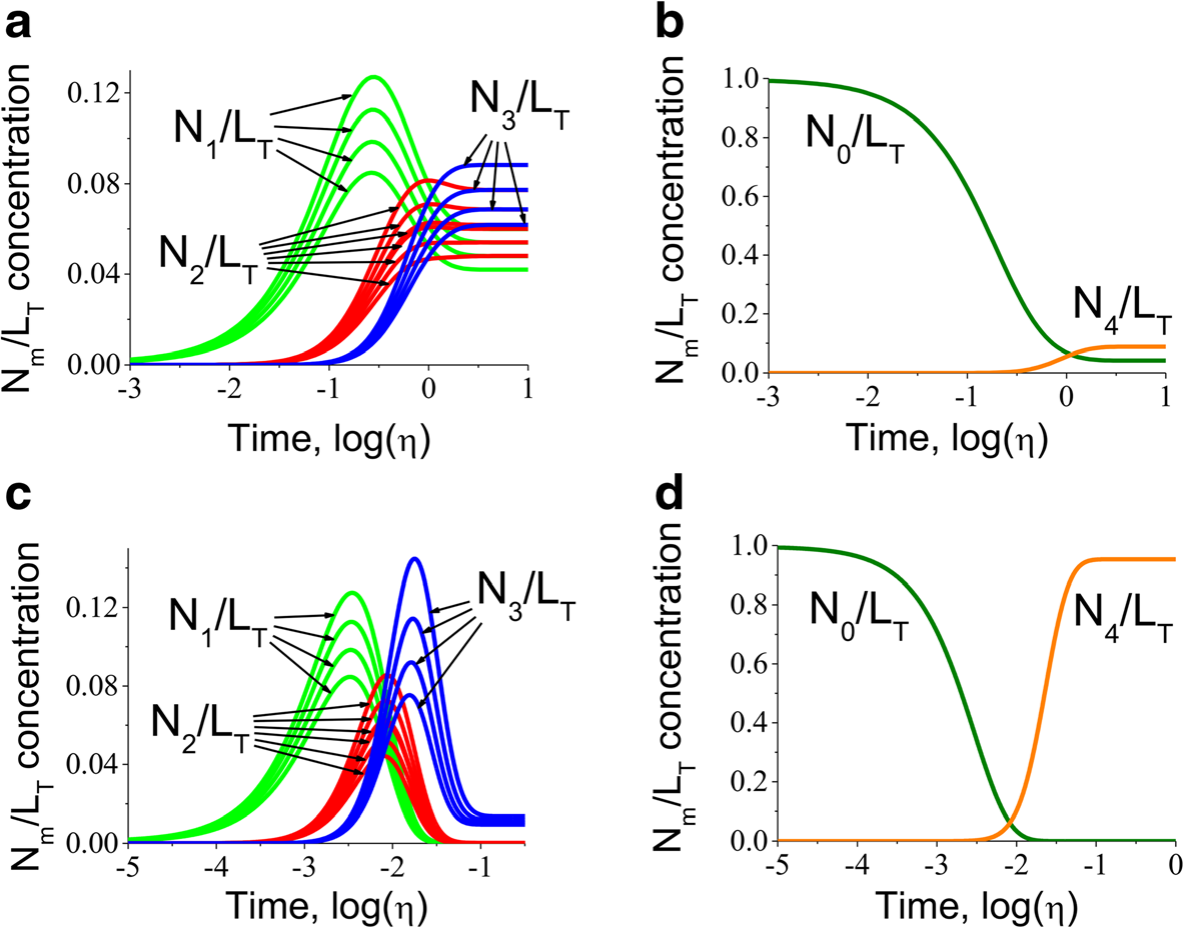
Kinetics predictions for multisite protein species with marginally different association constants. The kinetics of multisite protein species was investigated for the intermediate (*N*_1_/*L*_*T*_, *N*_2_/*L*_*T*_ and *N*_3_/*L*_*T*_ in **a** as well as the apo- and fully bound conformations (*N*_0_/*L*_*T*_ and *N*_4_/*L*_*T*_ respectively in **b** in response to step change of ligand from *U*_0_/*K* = 0.001 to *U*_1_/*K* = 1.43 for marginally different association constants *h*_1_ = 1, *h*_2_ = 0.9, *h*_3_ = 0.8, *h*_4_ = 0.7 and the same dissociation constants *h*_1_^−^ = *h*_2_^−^ = *h*_3_^−^ = *h*_4_^−^ = 1. Similar analysis was also performed when step change was *U*_0_/*K* = 0.001, *U*_1_/*K* = 100 for apo- (**c**) and fully bound (**d**) forms. The calculations show that the final level of the multisite protein species are defined by the ligand concentration after the step change. It is very clear that the fully bound species are not saturated and most of the ligand is distributed among species bound to fewer ligands. However, step change application of ligand with much higher concentration from *U*_0_/*K* = 0.001 to *U*_1_/*K* = 100 for apo- (**c**) and fully bound (**d**) species demonstrate that the application of higher concentrations of ligand causes fully saturates the protein

**Fig. 6:**
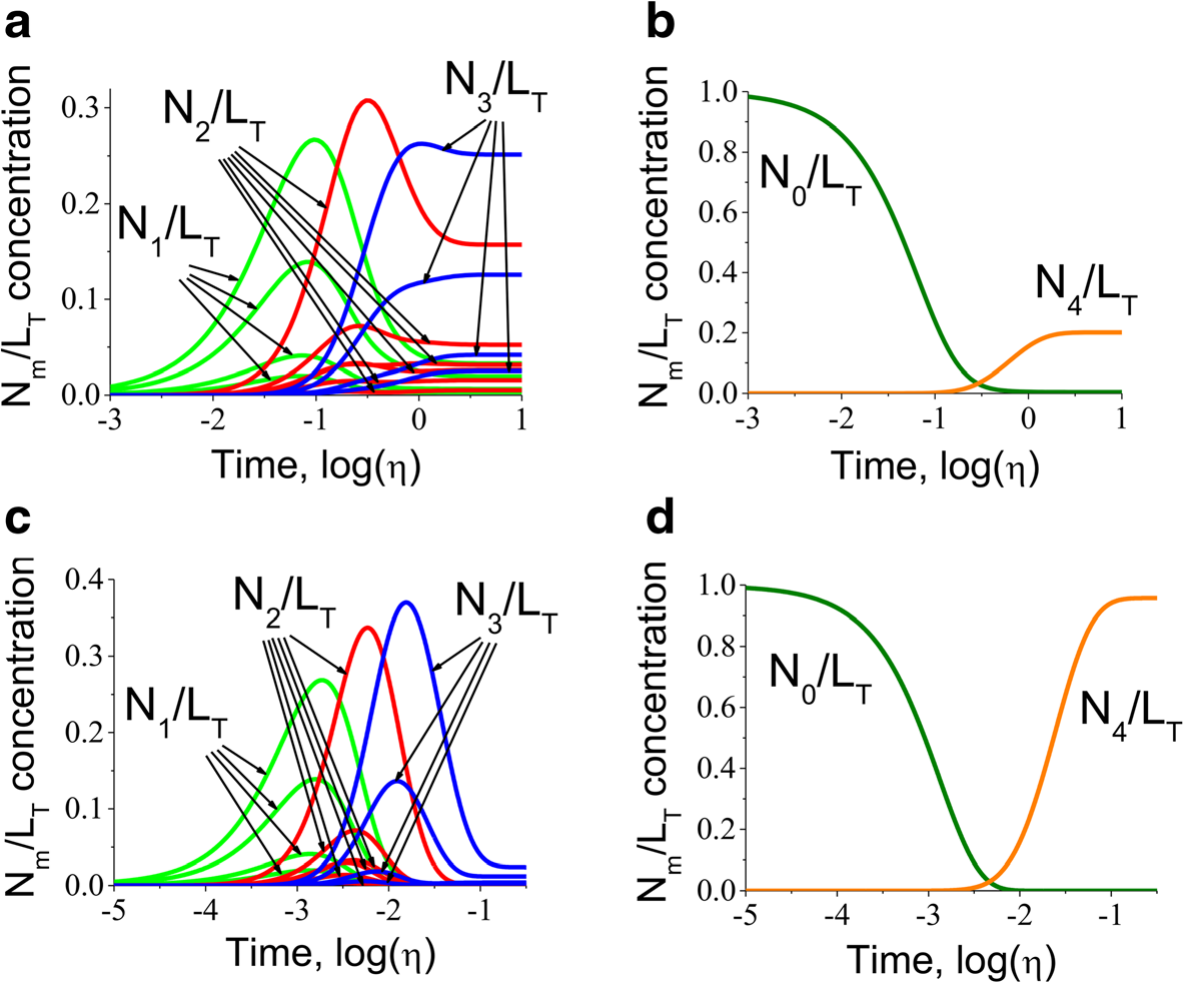
Kinetics of multisite protein species alterations for a protein with significantly different association constants. The kinetics of multisite protein species was investigated in response to step change of ligand from *U*_0_/*K* = 0.001 to *U*_1_/*K* = 8 and to *U*_1_/*K* = 400 for the intermediate (*N*_1_/*L*_*T*_, *N*_2_/*L*_*T*_ and *N*_3_/*L*_*T*_ in **a**, **c**) as well as apo- and fully bound conformations (*N*_0_/*L*_*T*_ and *N*_4_/*L*_*T*_ in **b**, **d**), respectively, in the case of significantly different association *h*_1_ = 1, *h*_2_ = 0.6, *h*_3_ = 0.2, *h*_4_ = 0.1 and the same dissociation constants *h*_1_^−^ = *h*_2_^−^ = *h*_3_^−^ = *h*_4_^−^ = 1. The comparison with the kinetics of the protein with slightly different association constants suggests that in this case the species acquire a degree of asynchronous dynamics

To investigate the impact of the dissociation constants of individual binding sites we employed the multisite protein model (please see subsection “Multisite proteins with four different ligand binding sites” in Methods) with marginally (Fig. 5) and significantly different association constants (Fig. 6). The main result that follows from the analysis of the intermediate conformation curves is that the affinities of the different binding sites mainly affect the magnitudes of corresponding protein conformation. For example, the conformation of a multisite protein corresponding to the one ligand bound state is present in lower concentration if the affinity of the binding centre is lower. However the overall shape of the concentration dependent profile has not changed. This property is very similar to the case of steady-state dependence on the ligand concentration. The only difference is that the bell shape dependence on time during the kinetic response is partially skewed. However, significant variation in affinities changes the magnitude, and also leads to the asynchronous kinetics of the intermediate conformations.

### The effects of cooperativity in Ca^2+^ binding to CaM

**Fig. 7:**
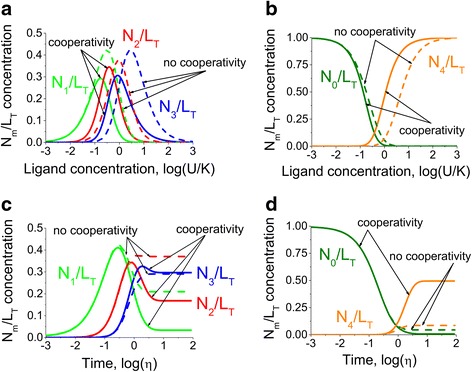
Comparative analysis of cooperative versus non-cooperative Ca^2+^ binding to CaM. Two mathematical models for Ca^2+^-Cam interactions are compared under the assumptions for the presence and absence of cooperative binding. The comparison between the two scenarios was performed under steady state conditions (**a**), (**b**) and in response to a step change in Ca^2+^ concentration (**c**), (**d**). The model predicts that the cooperativity influences the maximums of the concentrations for the intermediate forms (*N*_1_/*L*_*T*_, *N*_2_/*L*_*T*_ and *N*_3_/*L*_*T*_) as well as the steady-state levels of apo- (*N*_0_/*L*_*T*_) and fully saturated forms (*N*_4_/*L*_*T*_). However, the difference observed in the distribution of the conformation species in the presence and absence of the cooperative binding is quantitative while the overall shape of the distributions remains unchanged. Due to this finding the following model analysis was performed without cooperative binding assumptions

In order to investigate the influence of cooperativity, we chose a well-characterized protein, CaM, as the model object. The CaM protein contains two independent EF-hand globular domains, with two binding sites [1, 3, 5, 16, 17]. The sites within each of the domains cooperatively influence each other. It has been reported that cooperative binding occurs between two neighbouring sites within the N- and C- terminal domains of CaM [22, 53, 54]. Figure 7 shows the model predictions for CaM where we assume that the molecule has two independent domains, with two identical cooperative sites. In the first domain the affinity of one site changes from *K*_1_ = 0.9 μM to *K*_1_^*c*^ = 0.2 μM if the other site is occupied and in the second domain the affinity changes from *K*_2_ = 0.8 μM to *K*_2_^*c*^ = 0.1 μM (Eq. (28) in Methods) [22]. Figure 7a and b show the influence of cooperativity on the steady-state concentrations of CaM with certain number of bound sites. The presence of cooperativity shifts the dose-response characteristics along the ligand concentration axis and changes the magnitude of intermediate conformations allowing more developed selective regulation of the activity of CaM. The investigation of the dynamic properties of co-operativity in CaM (Fig. 7c and d) for intermediate, apo- and saturated species revealed that the cooperativity influences the magnitudes of time-dependent characteristics. The proposed model predicts that the cooperative binding leads to more pronounced selective effects for intermediate conformations and higher differences between the initial and steady-state levels for the apo- and saturated forms.

The results of the present analysis suggest that cooperativity plays an important role in the regulation of the activity of multisite proteins by allowing wider possibilities for selectivity. However, the presence of cooperativity leads to quantitative rather than qualitative changes in the system. The introduction of cooperative binding is crucial for the experimental data fitting but at the same time brings further complexity to the system, which does not necessarily lead to a better understanding of the underlying mechanisms. As a result of this, further analysis was carried out without considering this effect.

## The model for comparable ligand and protein concentrations

**Fig. 8:**
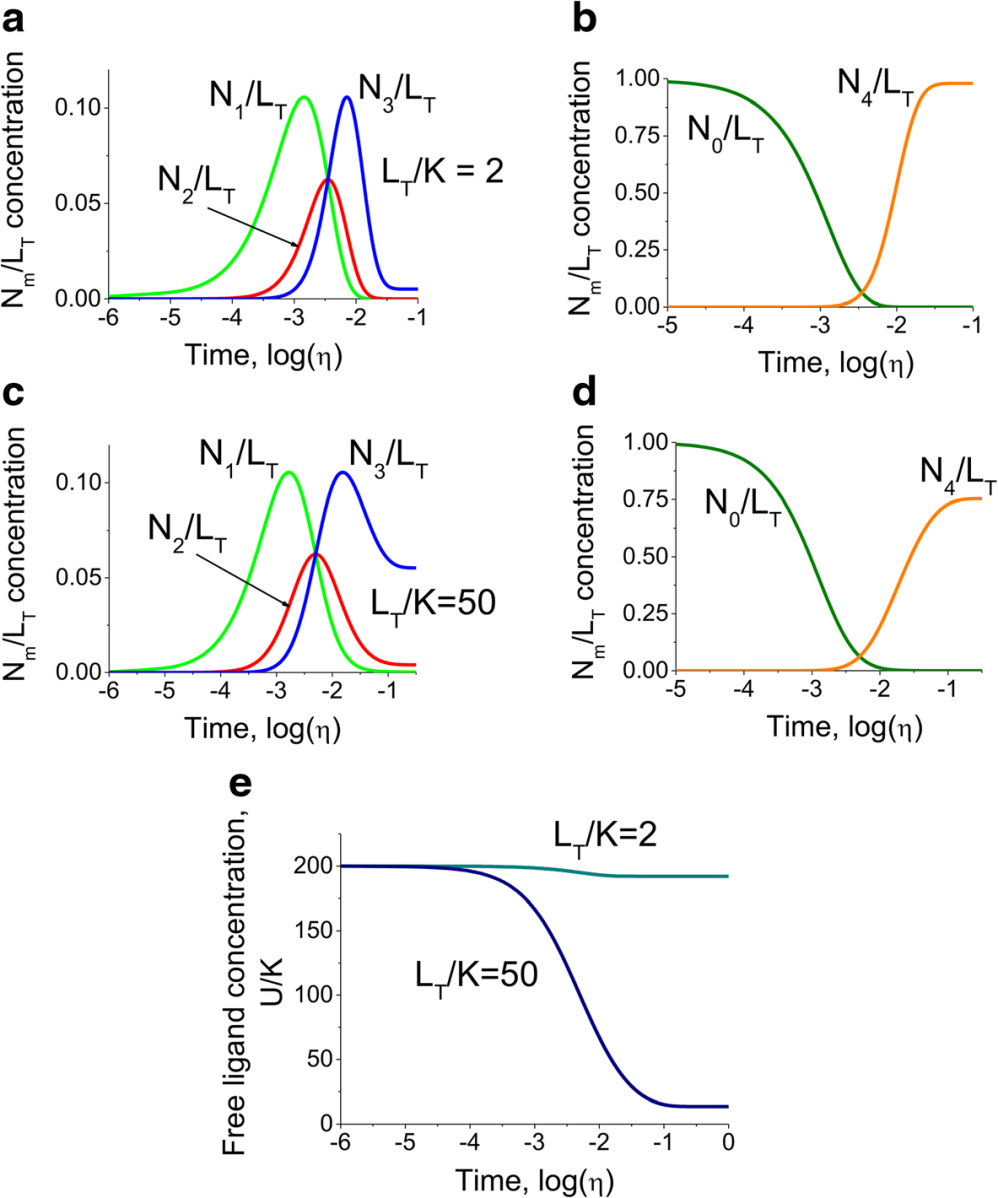
The comparison between the cases where the free ligand concentration is barely affected by interaction and exhausted as a result of buffering. The kinetics of multisite protein species alterations in response to step change in ligand concentration from *U*_*T*0_/*K* = 0.01 to *U*_*T*1_/*K* = 200 for two different ratios of the protein concentration to the affinities of the binding sites *L*_*T*_/*K* = 2 **a** for the intermediate species *N*_1_/*L*_*T*_, *N*_2_/*L*_*T*_ and *N*_3_/*L*_*T*_, **b** for the apo- and fully bound species *N*_0_/*L*_*T*_ and *N*_4_/*L*_*T*_ respectively) and *L*_*T*_/*K* = 50 **c** for the intermediate species *N*_1_/*L*_*T*_, *N*_2_/*L*_*T*_ and *N*_3_/*L*_*T*_, **d** for the apo- and fully bound species *N*_0_/*L*_*T*_ and *N*_4_/*L*_*T*_ respectively). The model predicts that due to the lack of available ligand and buffering by the multisite protein in the case of limited amount of ligand, the multisite protein is unable to become fully saturated after the step change in ligand, and the majority of the ligand becomes distributed among the intermediate species. **e.** The comparison of the dynamics of free ligand concentration *U*/*K* after step change in ligand. The amount of available ligand is barely altered for *L*_*T*_/*K* = 2, and exhausted when the ratio of total protein concentration to the binding constant is *L*_*T*_/*K* = 50

The previous sections considered the physiological case where the ligand concentration was above saturation level meaning that the ligand-multisite protein interactions did not affect the availability of the ligand. Here the case when the amount of ligand is limited is considered. This can occur in cases when the ligand concentration level is comparable to the multisite protein availability. The model predictions show the relative redistribution of the ligand in the free and bound states. The responses of a multisite protein with four identical binding sites to ligand concentration step change (Eqs. (44) and (45) in Methods) for two different protein concentrations, *L*_*T*_/*K* = 2 and *L*_*T*_/*K* = 50, were studied (Fig. 8). Instead of considering absolute ligand concentrations, the approach considered ratios of the ligand concentration to the affinities of the binding sites. A borderline case where the free ligand concentration is barely affected by interaction was considered (Fig. 8a and b) as well as a smaller total ligand concentration where the free ligand is nearly exhausted as a result of buffering by the multisite protein (Fig. 8c and d). The comparison of the free ligand concentration (Eqs. (43) and (44) in Methods) for these two cases is shown in Fig. 8e. The model predicts that the strongest effect of the ligand availability can be observed for the multisite protein conformations with three and four (fully saturated) bound ligands. A possible explanation for this phenomenon may be that the multisite protein conformations, which form complexes with smaller number of ligand molecules by definition, do not require significant amount of ligand and as a result are not strongly affected under conditions when the free ligand is limited. Whereas the multisite protein interactions with the larger number of ions occur after the significant amount of ligand is “used up” to form the intermediate conformations, however is still required for conformations with larger number of ions. As a result the final levels of the conformations with three and four ions are affected. It can also be seen from Fig. 8 that the shapes of the intermediate conformations time lines are skewed.

**Fig. 9:**
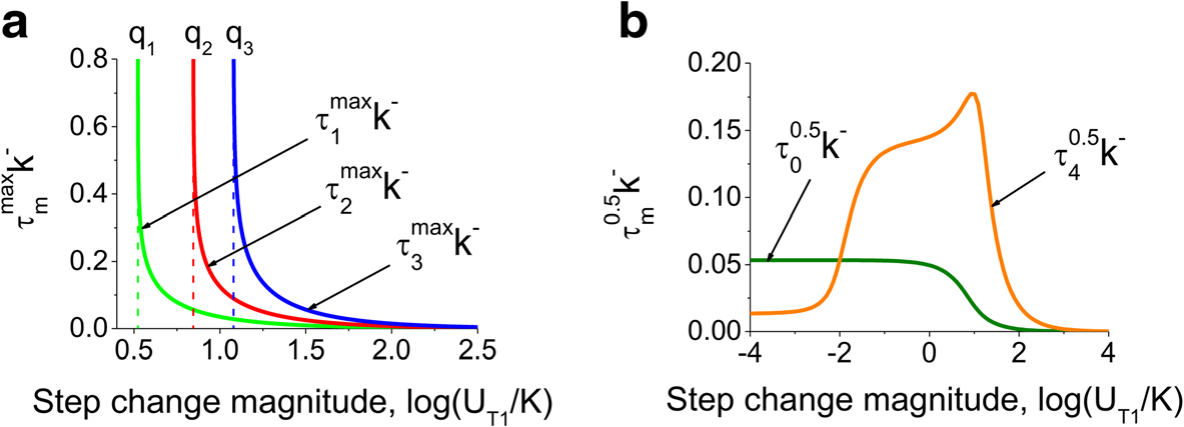
Model predictions for the time required for multisite protein conformations to reach their maximal and half growth concentrations. The non-dimensional characteristic times, *τ*_*m*_^*max*^ *k* ^−^ (*m* = 1, 2, 3) for intermediate (**a**), *τ*_0_^0.5^ *k* ^−^ for apo- and *τ*_4_^0.5^ *k* ^−^ for fully bound (**b**) multisite protein conformations are shown as a function of the step change ligand concentration from *U*_*T*0_/*K* = 0.1 to *U*_*T*1_/*K* and *L*_*T*_/*K* = 3

Figure 9a shows the model predictions for the characteristic time required for intermediate protein conformations with one, two and three bound ligands to reach their highest concentrations in response to a step change in ligand concentration (Eq. (50) in Methods). The model predicts that the characteristic time *τ*_0_^0.5^, required for the apo- form to reach its half growth level monotonically decreases with the increase of the total ligand concentration. However, the characteristic time constant *τ*_4_^0.5^, which represents the saturated conformation reveals a distorted bell shaped dependence on ligand concentration (Fig. 9b and Eqs. (52) in Methods). This bell shaped dependence, which was also observed in Fig. 3d, can also be explained by the presence of intermediate conformations. The distortion of the bell shape in Fig. 9b appears to be due to the ligand consumption that is included into the consideration in this section and was not considered in Fig. 3d. This result may be significant for the dynamics of CaM activation as our model predicts that with an increase of the total ligand concentration, the limited amount of ligand leads to an additional increase of *τ*_4_^0.5^ compared to the case without the ligand consumption. The model, therefore, predicts possible transient differences in multisite protein signal transduction in response to fast transient kinetics of multisite proteins.

## Discussion

In this paper we analysed multisite protein kinetics in response to rapid changes in ligand concentrations. The model for multisite protein kinetics for variable number of bound ligands was developed for two physiological cases: when the concentration of ligand is much higher [55, 56] and when it is comparable with the concentration of the multisite protein [57]. The results obtained by the proposed model allow for an accurate interpretation of the experimental data for the concentration of multisite proteins such as CaM, TnC, CaN and other Ca^2+^ dependent secondary messengers regulated by Ca^2+^ ions. The kinetic effects in response to ligand binding [13, 58, 59], required for understanding of pathway regulation, can be interpreted by the presented model.

The approach developed in this project is applicable to a number of areas of kinetic experiments. One of them is widely used techniques to study chemical kinetics using the pressure jump technique [12, 60, 61]. According to our model, the effects induced by rapid change in pressure leading to the change in protein-ligand interactions [12, 60, 61], can be interpreted and explained by the alteration in the affinities of the binding constants of multisite proteins. The time dynamics of the individual multisite protein species can offer new insights into the underlying biophysical mechanism of ligand-protein interactions in response to fast change.

Our model shows that the concentration of intermediate conformations as a function of time represents skewed bell shapes. We found that as the protein concentration rises, free ligand concentration becomes exhausted [13]. This result is consistent with experimentally observed Ca^2+^-CaM-dependent inhibition of myosin motor function [62]. The results obtained by this model increase our understanding of differential activation of protein phosphatase 2B (PP2B) [59, 63] and calcium/calmodulin-dependent protein kinase II (CaMKII) [64, 65] kinetics. PP2B binding increases the affinity of CaM for its targets [59] and, therefore, is likely be activated by low amounts of calcium.

The presented model offers a new tool for the interpretation of transient kinetics experiments performed by the flash photolysis and stopped-flow techniques [66–68]. Availability of a caged analogue of the necessary reactant is considered as one of the biggest problems of flash photolysis [66], which can be overcome by employing the proposed model with the low amount of protein. A less obvious area of application for this methodology is the kinetics of proteins with multiple phosphorylation sites [69–72]. This work shows that the highly versatile intracellular multifunctionality of multisite proteins is achieved not only by the order of ligand-protein interaction and the number of bound ligands, but also by temporal regulation.

## Conclusions

The developed in this study models for the kinetics of the multisite ligand-receptor binding for the two physiological cases, where the ligand concentration is abundant and comparable with the protein concentration, make universal predictions for the temporal distribution of multisite protein conformations in complex with variable numbers of ligands. The strongest effect of the ligand availability is observed for the multisite protein conformations with larger numbers of bound ligands. The two models show that the concentration of individual multisite protein conformations changes with time nonlinearly and that the temporal distribution for the concentrations of the intermediate conformations represents skewed bell shapes. The models derive the characteristic times and the dynamics for the kinetic responses elicited by a ligand concentration change as a function of ligand concentration and the number of ligand binding sites. The developed models allow for a novel and accurate interpretation of concentration and pressure jump-dependent kinetic experiments. Our models are applied to study the kinetics of calmodulin, however the models also provide universal predictions and allow us to extend the understanding of a large number of multisite binding-regulated circuits and the mechanisms underlying dynamic ligand-dependent regulation of multisite proteins.

## Methods

### Kinetic properties of multisite proteins with n binding sites

Here, we describe mathematical equations used to describe dynamic interactions of ligand molecules with multisite proteins. The model described in this section extends our previous analysis for multisite protein interactions under steady-state conditions [22–24].

### Multisite protein with independent ligand binding sites

The kinetic scheme for such interaction can be represented as follows:

1 $$ {L}_i^0+U\underset{k_i^{-}}{\overset{k_i^{+}}{\rightleftarrows }}{L}_i^1,\ i=0,\dots, n-1, $$

where *L*_*i*_^0^ is the *i*th binding site of the multisite protein in unbound state, *U* is a ligand molecule, *L*_*i*_^1^ is the *i*th binding site of the multisite protein being occupied, *k*_*i*_^+^ and *k*_*i*_^−^ are the association and dissociation rates respectively.

The probabilities for the *i*th binding site to be not occupied or occupied as a function of ligand concentration *U* are given by:

2 $$ \begin{array}{l}{p}_i^0(U)=\frac{K_i}{K_i+U},\\ {}{p}_i^1(U)=\frac{U}{K_i+U},\end{array} $$

where $$ {K}_i=\frac{k_i^{-}}{k_i^{+}}. $$

There are two possible states of a binding site: occupied or not occupied. Since the number of sites in the molecule is *n*, there are 2^*n*^ possible molecular forms, i.e. the states characterized by combinations of bound and free sites.

The probability for a multisite protein with independent binding sites to be in a particular molecular form is given by multiplications of probabilities of ligand binding at each site:

3 $$ {P}_j(U)={\displaystyle \prod_{i=0}^{n-1}{p}_i^{c_i(j)}}(U),\ j=0,\dots, {2}^n-1, $$

where $j={\displaystyle \sum_{i=0}^{n-1}{2}^i{c}_i(j)}$ is the number of possible molecular form, *c*_*i*_(*j*) = 0 or *c*_*i*_(*j*) = 1 for free or occupied binding site, respectively.

## The kinetics of multisite protein interactions with abundant ligand concentrations (U_T_ > > L_T_)

In this case we assume the ligand concentration only changes at a given time point (*t* = 0) in a step fashion from *U*_0_ to *U*_1_ and remains constant otherwise.

The probability of a multisite protein being occupied by ligand molecules as it was shown in Eq. (3) as a function of time can be reformulated as follows:

4 $$ {P}_j\left(U,t\right)={\displaystyle \prod_{i=0}^{n-1}{p}_i^{c_i(j)}}\left(U,t\right),\ j=0,\dots, {2}^n-1. $$

${p}_i^{c_i(j)}\left(U,t\right)$ can be determined by considering the original set of ordinary differential equations for single site interaction of a protein with a ligand according to Eq. (1) [24]:

5 $$ \begin{array}{l}\frac{d{L}_i^0\left(U,t\right)}{dt}=-{k}_i^{+}\cdot {L}_i^0\left(U,t\right)\cdot {U}_1+{k}_i^{-}\cdot {L}_i^1\left(U,t\right),\\ {}\frac{d{L}_i^1\left(U,t\right)}{dt}={k}_i^{+}\cdot {L}_i^0\left(U,t\right)\cdot {U}_1-{k}_i^{-}\cdot {L}_i^1\left(U,t\right),\\ {}{L}_i^0\left(U,t\right)+{L}_i^1\left(U,t\right)={L}_T,\end{array} $$

where *L*_*i*_^0^(*U*, *t*) is the concentration of a free binding site, *L*_*i*_^1^(*U*, *t*) is the concentration of a bound ligand molecule and *L*_*T*_ is the total number of the protein molecules.

Here we use steady-state solutions ${L}_i^0(U)={L}_T\frac{K_i}{K_i+U}$ and ${L}_i^1(U)={L}_T\frac{U}{K_i+U}$ as initial conditions for the ligand concentration jump from *U*_0_ to *U*_1_. A particular solution for the system of differential Eqs. (5) in response to the ligand concentration shift from *U*_0_ to *U*_1_ is given by:

6 $$ \begin{array}{l}{L}_i^0\left(U,t\right)={L}_T\cdot \left(\frac{K_i}{K_i+{U}_1}-\left(\frac{K_i}{K_i+{U}_1}-\frac{K_i}{K_i+{U}_0}\right)\cdot \exp \left(-\frac{t}{\tau \left({U}_1\right)}\right)\right),\\ {}{L}_i^1\left(U,t\right)={L}_T\cdot \left(\frac{U_1}{K_i+{U}_1}-\left(\frac{U_1}{K_i+{U}_1}-\frac{U_0}{K_i+{U}_0}\right)\cdot \exp \left(-\frac{t}{\tau \left({U}_1\right)}\right)\right),\end{array} $$

where $$ \tau \left({U}_1\right)=\frac{K_i}{k_i^{-}\cdot \left({U}_1+{K}_i\right)},{K}_i=\frac{k_i^{-}}{k_i^{+}}. $$

The normalisation of the solution (6) by the total protein concentration allows the definition of probability of the *i*th binding site to be in an occupied *p*_*i*_^1^(*U*, *t*) or unoccupied *p*_*i*_^0^(*U*, *t*) state, respectively, at a given ligand concentration:

7 $$ \begin{array}{l}{p}_i^0\left(U,t\right)={p}_i^0\left({U}_1\right)-\left({p}_i^0\left({U}_1\right)-{p}_i^0\left({U}_0\right)\right)\cdot \exp \left(-\frac{t}{\tau \left({U}_1\right)}\right),\\ {}{p}_i^1\left(U,t\right)={p}_i^1\left({U}_1\right)-\left({p}_i^1\left({U}_1\right)-{p}_i^1\left({U}_0\right)\right)\cdot \exp \left(-\frac{t}{\tau \left({U}_1\right)}\right).\end{array} $$

At any given time the total concentration of the protein, *L*_*T*_, is conserved and the sum of the probabilities (7) equals 1:

8 $$ \begin{array}{l}{L}_i^0\left(U,t\right)+{L}_i^1\left(U,t\right)={L}_T,\\ {}{p}_i^0\left(U,t\right)+{p}_i^1\left(U,t\right)=1\end{array} $$

In the most general case, the concentration of the *j*th molecular form, *M*_*j*_(*U*, *t*), of a molecule with *n* different binding sites in response to a step in ligand concentration is given by the product of probabilities (7) according to Eq. (4):

9 $$ {M}_j\left(U,t\right)={L}_T\cdot {\displaystyle \prod_{i=0}^{n-1}{p}_i^{c_i(j)}}\left(U,t\right),\ j=0,\dots, {2}^n-1, $$

where *c*_*i*_(*j*) = 0 or *c*_*i*_(*j*) = 1 for free or occupied *i*th binding site respectively.

The probabilities for individual molecular forms in steady-state can be obtained from Eq. (9) by setting *t* → ∞:

10 $$ {M}_j(U)={L}_T\cdot {\displaystyle \prod_{i=0}^{n-1}{p}_i^{c_i(j)}}(U),\ j=0,\dots, {2}^n-1, $$

The kinetics of the amount of ligand bound to a multisite protein with *n* binding sites can be written as follows:

11 $$ S\left(U,t\right)={\displaystyle \sum_{j=0}^{2^n-1}{M}_j\left(U,t\right){\displaystyle \sum_{i=0}^{n-1}{c}_i(j)}}. $$

### Multisite proteins with identical ligand binding sites

The multisite proteins that contain *n* identical binding sites with equilibrium dissociation constant equal $K=\frac{k^{-}}{k^{+}}$ can then be considered. The steady-state concentration of one of the molecular forms of multisite protein with *n* identical binding sites for *m* bound sites, as a function of ligand concentration [22, 24] is given by:

12 $$ {N}_m(U)={L}_T\cdot \frac{U^m\cdot {K}^{n-m}}{{\left(K+U\right)}^n},\ m=0,1,\dots, n. $$

For intermediate forms the *N*_1_(*U*), …, *N*_*n* − 1_(*U*) species dependence on ligand concentration represents a bell shape with one molecular form “magnitude” at a particular ligand concentration *U*_*m*_^*max*^.

Differentiating Eq. (12) with respect to *U* and solving *dN*/*dU* = 0 for *U* gives:

13 $$ {U}_m^{max}=\frac{K\cdot m}{n-m},\ m=1,2,\dots, n-1. $$

The magnitudes *N*_*m*_^*max*^ of the intermediate conformations *N*_*m*_ corresponding to *U*_*m*_^*max*^ values are:

14 $$ {N}_m^{max}={L}_T\cdot \frac{m^m}{n^n}{\left(n-m\right)}^{n-m},\ m=1,2,\dots, n-1. $$

The multisite protein conformations in the apo-, *N*_0_, and in the fully saturated states, *N*_*n*_, would reach their maximum that equal to the total multisite protein concentration *L*_*T*_ under conditions of very low and very high ligand concentrations, respectively.

Equation (12) can be used to estimate the half maximal effective ligand concentration (*EC*_50_), *U*_0_^0.5^ and *U*_*n*_^0.5^, for the apo- and saturated multisite protein conformations respectively, when the protein species equal 50 % of the total concentration *L*_*T*_:

15 $$ \begin{array}{l}{U}_0^{0.5}=K\cdot \frac{1-\sqrt[n]{0.5}}{\sqrt[n]{0.5}},\\ {}{U}_n^{0.5}=K\cdot \frac{\sqrt[n]{0.5}}{1-\sqrt[n]{0.5}}\approx K\cdot \left(1.44\cdot n-0.44\right).\end{array} $$

It can be noted that the half maximal effective ligand concentration is equal to equilibrium dissociation constant (*EC*_50_ = *K*) in proteins with only one binding site (*n* = 1).

Equations (15) can be solved with respect to *n*:

16 $$ n=0.693\cdot {\left( \ln \left(\frac{K+{U}_n^{0.5}}{U_n^{0.5}}\right)\right)}^{-1} $$

Equation (11) can be used to derive the full amount of ligand bound to multisite protein for *U* = *K*:

17 $$ S\left(K,t\right)=\frac{1}{4}\cdot S\left({U}^{sat},t\right), $$

where *U*^*sat*^ is the ligand concentration for the case when all binding sites are occupied. The amount of bound ligand for *U* = *U*^*sat*^ is given by *S*(*U*^*sat*^, *t*) = *n* ⋅ *L*_*T*_ and for *U* = *K* is given by

18 $$ S\left(K,t\right)=\frac{L_T}{2^n}\cdot \frac{1}{2}\cdot n\cdot {2}^{n-1}. $$

The dynamic alterations of intermediate conformation *N*_*m*_(*U*, *t*) in response to ligand concentration jump from *U*_0_ to *U*_1_ according to Eqs. (6) and (7) are given by:

19 $$ \begin{array}{l}{N}_m\left(U,t\right)={L}_T\cdot {\left({p}^1\left(U,t\right)\right)}^m\cdot {\left({p}^0\left(U,t\right)\right)}^{n-m},\\ {}{p}^0\left(U,t\right)=\frac{K}{K+{U}_1}-\left(\frac{K}{K+{U}_1}-\frac{K}{K+{U}_0}\right)\cdot \exp \left(-\frac{t}{\tau \left({U}_1\right)}\right),\\ {}{p}^1\left(U,t\right)=\frac{U_1}{K+{U}_1}-\left(\frac{U_1}{K+{U}_1}-\frac{U_0}{K+{U}_0}\right)\cdot \exp \left(-\frac{t}{\tau \left({U}_1\right)}\right),\\ {}\tau \left({U}_1\right)=\frac{K}{k^{-}\cdot \left({U}_1+K\right)}.\end{array} $$

Differentiating Eq. (19) with respect to *t* and solving for *dN*_*m*_/*dt* = 0 yields the time *τ*_*m*_^*max*^ when the multisite protein forms *N*_*m*_ with *m* bound molecules of ligand reach their maximal values *N*_*m*_^*max*^:

20 $$ {\tau}_m^{max}=\tau \left({U}_1\right)\cdot \ln \left(\frac{n\cdot K\cdot \left({U}_1-{U}_0\right)}{\left(K+{U}_0\right)\cdot \left(\left(n-m\right)\cdot {U}_1-m\cdot K\right)}\right),\ m=1,2,\dots, n-1. $$

The substitution of *τ*_*m*_^*max*^ into Eq. (19) gives the maximal values of intermediate multisite protein conformations reached at *τ*_*m*_^*max*^:

21 $$ {N}_m^{max}={L}_T\cdot \frac{m^m}{n^n}{\left(n-m\right)}^{n-m},\ m=1,2,\dots, n-1. $$

Comparison of Eqs. (14) and (21) suggests that the steady-state maximum values of intermediate multisite protein conformations steady-state are similar to those transiently reached during the dynamic response to the step in ligand concentration.

According to Eq. (19) *N*_*m*_(*U*, *t*) for the apo- and fully saturated forms when *t* = 0:

22 $$ \begin{array}{l}{N}_0\left(U,0\right)={L}_T\cdot {\left(\frac{K}{K+{U}_0}\right)}^n,\\ {}{N}_n\left(U,0\right)={L}_T\cdot {\left(\frac{U_0}{K+{U}_0}\right)}^n.\end{array} $$

According to Eq. (12) steady state levels for the apo- and fully saturated forms when *t* → ∞:

23 $$ \begin{array}{l}{N}_0\left(U,\infty \right)={L}_T\cdot {\left(\frac{K}{K+{U}_1}\right)}^n,\\ {}{N}_n\left(U,\infty \right)={L}_T\cdot {\left(\frac{U_1}{K+{U}_1}\right)}^n.\end{array} $$

Equation (19) is further used to define the time, *τ*_0_^0.5^, required for the apo- form, *N*_0_, to reach half of the growth concentration $\frac{N_0\left(U,\infty \right)+{N}_0\left(U,0\right)}{2}$ and the time period, *τ*_*n*_^0.5^, required for the fully saturated protein species to gain half of the growth concentration $\frac{N_n\left(U,\infty \right)+{N}_n\left(U,0\right)}{2}$ (Fig. 3):

24 $$ \begin{array}{l}{\tau}_0^{0.5}=\tau \left({U}_1\right)\cdot \ln \left(K\cdot \frac{U_1-{U}_0}{\left(K+{U}_0\right)\cdot \left(\left(K+{U}_1\right)\cdot {\left(0.5\cdot \left({\left(\frac{K}{K+{U}_0}\right)}^n+{\left(\frac{K}{K+{U}_1}\right)}^n\right)\right)}^{\frac{1}{n}}-K\right)}\right),\\ {}{\tau}_n^{0.5}=\tau \left({U}_1\right)\cdot \ln \left(K\cdot \frac{U_1-{U}_0}{\left(K+{U}_0\right)\cdot \left({U}_1-\left(K+{U}_1\right)\cdot {\left(0.5\cdot \left({\left(\frac{U_0}{K+{U}_0}\right)}^n+{\left(\frac{U_1}{K+{U}_1}\right)}^n\right)\right)}^{\frac{1}{n}}\right)}\right).\end{array} $$

### Multisite proteins with four identical ligand binding sites

This study next considered the kinetic properties of a protein with 4 binding sites. This allows 2^4^ = 16 molecular forms, each with potentially unique biochemical properties. There are 4 possible combinations of protein species bound to one or to three ligands, and 6 possible distinct molecular forms with two sites occupied. In the previous work we described the steady-state dependence of the individual multisite conformations on ligand concentration [22–24]. Here we analyse the kinetic transition of the individual species concentrations in response to the step in ligand concentration.

The dynamical alterations of intermediate conformation *N*_*m*_(*U*, *t*) in response to a step in ligand concentration from *U*_0_ to *U*_1_ according to Eq. (19) are given by:

25 $$ \begin{array}{l}{N}_m\left(u,\eta \right)={L}_T\cdot {\left({p}^1\left(u,\eta \right)\right)}^m\cdot {\left({p}^0\left(u,\eta \right)\right)}^{4-m},\\ {}{p}^0\left(u,\eta \right)=\frac{1}{1+{u}_1}-\left(\frac{1}{1+{u}_1}-\frac{1}{1+{u}_0}\right)\cdot \exp \left(-\eta \cdot \left({u}_1+1\right)\right),\\ {}{p}^1\left(u,\eta \right)=\frac{u_1}{1+{u}_1}-\left(\frac{u_1}{1+{u}_1}-\frac{u_0}{1+{u}_0}\right)\cdot \exp \left(-\eta \cdot \left({u}_1+1\right)\right),\end{array} $$

where: ${u}_0=\frac{U_0}{K}$, ${u}_1=\frac{U_1}{K}$ are non-dimensional ligand concentrations, and *η* = *t* ⋅ *k*^−^ is non-dimensional time. *k*^−^ and $K=\frac{k^{-}}{k^{+}}$ are the dissociation and equilibrium dissociation constants for ligand binding, respectively.

The maximum values of *N*_*m*_^*max*^ are reached at the following ligand concentrations: ${u}_1^{max}=\frac{K}{3}$, *u*_2_^*max*^ = *K*, *u*_3_^*max*^ = 3*K*, for the multisite protein species with one, two and three bound ions, respectively according to Eq. (13) and equal *N*_1_^*max*^ = 0.105*L*_*T*_, *N*_2_^*max*^ = 0.063*L*_*T*_, *N*_3_^*max*^ = 0.105*L*_*T*_, according to Eq. (14).

Differentiating Eq. (25) with respect to *η* and solving *dN*_*m*_/*dη* = 0 for *η* gives the non-dimensional time *η*_*m*_^*max*^ = *τ*_*m*_^*max*^*k*^−^ when the intermediate species reach their maximum:

26 $$ \begin{array}{l}{\eta}_1^{max}={\tau}_1^{max}{k}^{-}=\frac{1}{u_1+1} \ln \left(\frac{4\cdot \left({u}_1-{u}_0\right)}{\left(1+{u}_0\right)\cdot \left(3{u}_1-1\right)}\right),\\ {}{\eta}_2^{max}={\tau}_2^{max}{k}^{-}=\frac{1}{u_1+1} \ln \left(\frac{2\cdot \left({u}_1-{u}_0\right)}{\left(1+{u}_0\right)\cdot \left({u}_1-1\right)}\right),\\ {}{\eta}_3^{max}={\tau}_3^{max}{k}^{-}=\frac{1}{u_1+1} \ln \left(\frac{4\cdot \left({u}_1-{u}_0\right)}{\left(1+{u}_0\right)\cdot \left({u}_1-3\right)}\right).\end{array} $$

Equations (26) suggest that *η*_1_^*max*^, *η*_2_^*max*^ and *η*_3_^*max*^ are undefined when ${u}_1<\frac{1}{3},\ {u}_1<1\ \mathrm{and}\ {u}_1<3,$ respectively (*q*_1_, *q*_2_ and *q*_3_ asymptotes). Under these conditions, the intermediate species do not reach the maximum in response to ligand step, instead their relative number increase in a monotonous manner.

### Multisite proteins with four different ligand binding sites

In this section we analyse the kinetic properties of a multisite protein with four different binding sites. The steady-state analysis can be found in the authors previous investigation [22, 24].

It is assumed that all association *k*_1_^+^, *k*_2_^+^, *k*_3_^+^, *k*_4_^+^ and dissociation *k*_1_^−^, *k*_2_^−^, *k*_3_^−^, *k*_4_^−^ rates are unique for each binding centre. Then assuming for example that *k*_1_^+^ > *k*_2_^+^ > *k*_3_^+^ > *k*_4_^+^ and *k*_1_^−^ = *k*_2_^−^ = *k*_3_^−^ = *k*_4_^−^. The non-dimensional concentration $u=\frac{U}{K}$ and non-dimensional constants *h*_1_ = 1, ${h}_2=\frac{k_2^{+}}{k_1^{+}}$, ${h}_3=\frac{k_3^{+}}{k_1^{+}}$, ${h}_4=\frac{k_4^{+}}{k_1^{+}}$, *h*_1_^−^ = 1, ${h}_2^{-}=\frac{k_2^{-}}{k_1^{-}}=1$, ${h}_3^{-}=\frac{k_3^{-}}{k_1^{-}}=1$, ${h}_4^{-}=\frac{k_4^{-}}{k_1^{-}}=1$ can be introduced._._

The set of Eqs. (25) can then be employed to calculate the dynamics of the multisite protein conformations bound to a different number of ligand molecules.

### Multisite proteins with two pairs of cooperative binding sites

The molecule contains two independent domains *A* and *B*, with two identical cooperative binding sites. The domain *A* is described as follows:

27 $$ \begin{array}{l}\kern0.5em {A}_{00}+U\underset{k_1^{-}}{\overset{k_1^{+}}{\rightleftarrows }}{A}_{10}\\ {}\ {A}_{00}+U\underset{k_1^{-}}{\overset{k_1^{+}}{\rightleftarrows }}{A}_{01}\\ {}\ {A}_{10}+U\underset{k{c}_1^{-}}{\overset{k{c}_1^{+}}{\rightleftarrows }}{A}_{11}\\ {}{A}_{01}+U\underset{k{c}_1^{-}}{\overset{k{c}_1^{+}}{\rightleftarrows }}{A}_{11}\end{array} $$

The ODEs for the scheme (27) is given by:

28 $$ \begin{array}{l}\frac{d{A}_{00}\left(U,t\right)}{dt}=-2{k}_1^{+}\cdot {A}_{00}\left(U,t\right)\cdot U+{k}_1^{-}\cdot {A}_{10}\left(U,t\right)+{k}_1^{-}\cdot {A}_{01}\left(U,t\right),\\ {}\frac{d{A}_{10}\left(U,t\right)}{dt}={k}_1^{+}\cdot {A}_{00}\left(U,t\right)\cdot U-{k}_1^{-}\cdot {A}_{10}\left(U,t\right)-k{c}_1^{+}\cdot {A}_{10}\left(U,t\right)\cdot U+k{c}_1^{-}\cdot {A}_{11}\left(U,t\right),\\ {}\frac{d{A}_{01}\left(U,t\right)}{dt}={k}_1^{+}\cdot {A}_{00}\left(U,t\right)\cdot U-{k}_1^{-}\cdot {A}_{01}\left(U,t\right)-k{c}_1^{+}\cdot {A}_{01}\left(U,t\right)\cdot U+k{c}_1^{-}\cdot {A}_{11}\left(U,t\right),\\ {}\frac{d{A}_{11}\left(U,t\right)}{dt}=k{c}_1^{+}\cdot {A}_{01}\left(U,t\right)\cdot U+k{c}_1^{+}\cdot {A}_{10}\left(U,t\right)\cdot U-2k{c}_1^{-}\cdot {A}_{11}\left(U,t\right),\end{array} $$

The total number of species that follows from Eqs. (28) is given by:

29 $$ {A}_{00}\left(U,t\right)+{A}_{10}\left(U,t\right)+{A}_{01}\left(U,t\right)+{A}_{11}\left(U,t\right)={A}_T. $$

The steady-state solutions of the system (28) are given by:

30 $$ \begin{array}{l}{A}_{00}(U)={A}_T\cdot \frac{K_1\cdot {K}_1^c}{U^2+2\cdot {K}_1^c\cdot U+{K}_1\cdot {K}_1^c},\\ {}{A}_{10}(U)={A}_T\cdot \frac{U\cdot {K}_1^c}{U^2+2\cdot {K}_1^c\cdot U+{K}_1\cdot {K}_1^c},\\ {}{A}_{01}(U)={A}_T\cdot \frac{U\cdot {K}_1^c}{U^2+2\cdot {K}_1^c\cdot U+{K}_1\cdot {K}_1^c},\\ {}{A}_{11}(U)={A}_T\cdot \frac{U^2}{U^2+2\cdot {K}_1^c\cdot U+{K}_1\cdot {K}_1^c},\end{array} $$

where $$ {K}_1=\frac{k_1^{-}}{k_1^{+}} $$ and $$ {K}_1^c=\frac{k{c}_1^{-}}{k{c}_1^{+}} $$.

One can re-write Eqs. (30) as follows:

31 $$ \begin{array}{l}{a}_0(U)=\frac{K_1\cdot {K}_1^c}{U^2+2\cdot {K}_1^c\cdot U+{K}_1\cdot {K}_1^c},\\ {}{a}_1(U)=\frac{U\cdot {K}_1^c}{U^2+2\cdot {K}_1^c\cdot U+{K}_1\cdot {K}_1^c},\\ {}{a}_2(U)=\frac{U^2}{U^2+2\cdot {K}_1^c\cdot U+{K}_1\cdot {K}_1^c},\end{array} $$

where ${a}_0(U)=\frac{A_{00}(U)}{A_T},\ {a}_1(U)=\frac{A_{10}(U)}{A_T}=\frac{A_{01}(U)}{A_T},\ {a}_2(U)=\frac{A_{11}(U)}{A_T}$ are the probabilities for the domain to be in a particular conformation due to the bound ligand molecules.

The probabilities for the other domain, which also contains a pair of cooperative binding sites, are given by:

32 $$ \begin{array}{l}{b}_0(U)=\frac{K_2\cdot {K}_2^c}{U^2+2\cdot {K}_2^c\cdot U+{K}_2\cdot {K}_2^c},\\ {}{b}_1(U)=\frac{U\cdot {K}_2^c}{U^2+2\cdot {K}_2^c\cdot U+{K}_2\cdot {K}_2^c},\\ {}{b}_2(U)=\frac{U^2}{U^2+2\cdot {K}_2^c\cdot U+{K}_2\cdot {K}_2^c},\end{array} $$

These probabilities were derived for the two domains of molecule (Eqs. (31) and (32) respectively). The probabilities of the molecule to be in a certain conformation with 0, 1 or 2 bound ligands in each of the domains, are as follows:

33 $$ \begin{array}{l}{p}_{0,0}(U)={a}_0(U)\cdot {b}_0(U),\\ {}{p}_{0,1}(U)={a}_0(U)\cdot 2{b}_1(U),\\ {}{p}_{0,2}(U)={a}_0(U)\cdot {b}_2(U),\\ {}{p}_{1,0}(U)=2{a}_1(U)\cdot {b}_0(U),\\ {}{p}_{1,1}(U)=2{a}_1(U)\cdot 2{b}_1(U),\\ {}{p}_{1,2}(U)=2{a}_1(U)\cdot {b}_2(U),\\ {}{p}_{2,0}(U)={a}_2(U)\cdot {b}_0(U),\\ {}{p}_{2,1}(U)={a}_2(U)\cdot 2{b}_1(U),\\ {}{p}_{2,2}(U)={a}_2(U)\cdot {b}_2(U),\end{array} $$

where *p*_*i*,*j*_(*U*) is the probability of the protein conformation with *i* bound sites in the first domain and *j* bound sites in the other. We use the sum of probabilities for the case of 1 bound site in a domain since we assume that all the sites in each domain are identical.

The concentrations of the molecular forms of the protein with certain number of bound sites are given by:

34 $$ \begin{array}{l}{N}_0(U)={L}_T\cdot {p}_{0,0}(U),\\ {}{N}_1(U)={L}_T\cdot \left({p}_{0,1}(U)+{p}_{1,0}(U)\right),\\ {}{N}_2(U)={L}_T\cdot \left({p}_{0,2}(U)+{p}_{1,1}(U)+{p}_{2,0}(U)\right),\\ {}{N}_3(U)={L}_T\cdot \left({p}_{1,2}(U)+{p}_{2,1}(U)\right),\\ {}{N}_4(U)={L}_T\cdot {p}_{2,2}(U),\end{array} $$

One can rewrite Eq. (34) as follows:

35 $$ \begin{array}{l}\frac{N_0(U)}{L_T}={a}_0(U)\cdot {b}_0(U),\\ {}\frac{N_1(U)}{L_T}=2\cdot \left({a}_0(U)\cdot {b}_1(U)+{a}_1(U)\cdot {b}_0(U)\right),\\ {}\frac{N_2(U)}{L_T}={a}_0(U)\cdot {b}_2(U)+4\cdot {a}_1(U)\cdot {b}_1(U)+{a}_2(U)\cdot {b}_0(U),\\ {}\frac{N_3(U)}{L_T}=2\cdot \left({a}_1(U)\cdot {b}_2(U)+{a}_2(U)\cdot {b}_1(U)\right),\\ {}\frac{N_4(U)}{L_T}={a}_2(U)\cdot {b}_2(U),\end{array} $$

Next we find the kinetic solution of system (28) for *U* = *U*_1_:

36 $$ \begin{array}{l}\frac{d{A}_{00}\left(U,t\right)}{dt}=-2{k}_1^{+}\cdot {A}_{00}\left(U,t\right)\cdot {U}_1+{k}_1^{-}\cdot {A}_{10}\left(U,t\right)+{k}_1^{-}\cdot {A}_{01}\left(U,t\right),\\ {}\frac{d{A}_{10}\left(U,t\right)}{dt}={k}_1^{+}\cdot {A}_{00}\left(U,t\right)\cdot {U}_1-{k}_1^{-}\cdot {A}_{10}\left(U,t\right)-k{c}_1^{+}\cdot {A}_{10}\left(U,t\right)\cdot {U}_1+k{c}_1^{-}\cdot {A}_{11}\left(U,t\right),\\ {}\frac{d{A}_{01}\left(U,t\right)}{dt}={k}_1^{+}\cdot {A}_{00}\left(U,t\right)\cdot {U}_1-{k}_1^{-}\cdot {A}_{01}\left(U,t\right)-k{c}_1^{+}\cdot {A}_{01}\left(U,t\right)\cdot {U}_1+k{c}_1^{-}\cdot {A}_{11}\left(U,t\right),\\ {}\frac{d{A}_{11}\left(U,t\right)}{dt}=k{c}_1^{+}\cdot {A}_{01}\left(U,t\right)\cdot {U}_1+k{c}_1^{+}\cdot {A}_{10}\left(U,t\right)\cdot {U}_1-2k{c}_1^{-}\cdot {A}_{11}\left(U,t\right),\end{array} $$

## The kinetics of multisite protein interactions with constant ligand concentrations

In this section we consider the mechanism of multisite binding for the case when the total ligand concentration is conserved. Under this assumption the system of differential equations for ligand binding to a molecule with single binding site (5) needs to be complemented by the law of ligand conservation:

37 $$ \begin{array}{l}\frac{d{L}^0\left(U,t\right)}{dt}=-{k}^{+}\cdot {L}^0\left(U,t\right)\cdot U(t)+{k}^{-}\cdot {L}^1\left(U,t\right),\\ {}\frac{d{L}^1\left(U,t\right)}{dt}={k}^{+}\cdot {L}^0\left(U,t\right)\cdot U(t)-{k}^{-}\cdot {L}^1\left(U,t\right),\\ {}{L}^0\left(U,t\right)+{L}^1\left(U,t\right)={L}_T,\\ {}U(t)+{L}^1\left(U,t\right)={U}_T,\end{array} $$

where *L*^0^ is the concentration of the free site, *L*^1^ is the concentration of the occupied site, *U*_*T*_ and *L*_*T*_ are the total concentrations of ligand and protein molecules, respectively.

There is only one positive steady-state solution of the system (37):

38 $$ \begin{array}{l}{L}^0\left({U}_T\right)=\frac{K}{2}\cdot \left(\frac{L_T}{K}-\frac{U_T}{K}-1+F\left({U}_T\right)\right),\\ {}{L}^1\left({U}_T\right)=\frac{K}{2}\cdot \left(\frac{L_T}{K}+\frac{U_T}{K}+1-F\left({U}_T\right)\right),\\ {}U={U}_T-\frac{K}{2}\cdot \left(\frac{L_T}{K}+\frac{U_T}{K}+1-F\left({U}_T\right)\right),\end{array} $$

where $$ K=\frac{k^{-}}{k^{+}} $$ and $$ F\left({U}_T\right)=\sqrt{{\left(\frac{U_T}{K}-\frac{L_T}{K}\right)}^2+2\cdot \left(\frac{U_T}{K}+\frac{L_T}{K}\right)+1}. $$

We use the steady-state solutions (38) as initial conditions to find the particular solution. The solution of the system (37) in response to the ligand concentration jump from *U*_*T*0_ to *U*_*T*1_ is given by:

39 $$ \begin{array}{l}{L}^0\left({U}_T,t\right)={L}_T\cdot \left(\frac{K}{2\cdot {L}_T}\cdot \left(\frac{L_T}{K}-\frac{U_{T1}}{K}-1+F\left({U}_{T1}\right)\cdot \frac{C\left({U}_T\right)\cdot \exp \left(t\cdot {k}^{-}\cdot F\left({U}_{T1}\right)\right)+1}{C\left({U}_T\right)\cdot \exp \left(t\cdot {k}^{-}\cdot F\left({U}_{T1}\right)\right)-1}\right)\right),\\ {}{L}^1\left({U}_T,t\right)={L}_T\cdot \left(1-\frac{K}{2\cdot {L}_T}\cdot \left(\frac{L_T}{K}-\frac{U_{T1}}{K}-1+F\left({U}_{T1}\right)\cdot \frac{C\left({U}_T\right)\cdot \exp \left(t\cdot {k}^{-}\cdot F\left({U}_{T1}\right)\right)+1}{C\left({U}_T\right)\cdot \exp \left(t\cdot {k}^{-}\cdot F\left({U}_{T1}\right)\right)-1}\right)\right),\\ {}U(t)={U}_{T1}-{L}_T\cdot \left(1-\frac{K}{2\cdot {L}_T}\cdot \left(\frac{L_T}{K}-\frac{U_{T1}}{K}-1+F\left({U}_{T1}\right)\cdot \frac{C\left({U}_T\right)\cdot \exp \left(t\cdot {k}^{-}\cdot F\left({U}_{T1}\right)\right)+1}{C\left({U}_T\right)\cdot \exp \left(t\cdot {k}^{-}\cdot F\left({U}_{T1}\right)\right)-1}\right)\right).\end{array} $$

where $$ C\left({U}_T\right)=\frac{F\left({U}_{T0}\right)+F\left({U}_{T1}\right)+\frac{U_{T1}}{K}-\frac{U_{T0}}{K}}{F\left({U}_{T0}\right)-F\left({U}_{T1}\right)+\frac{U_{T1}}{K}-\frac{U_{T0}}{K}}. $$

The normalisation of the solution (39) by the total protein concentration allows the definition of probability of the binding site to be in an occupied *p*^1^(*U*_*T*_, *t*) or unoccupied *p*^0^(*U*_*T*_, *t*) state, respectively, at a given total ligand concentration:

40 $$ \begin{array}{l}{p}^0\left({U}_T,\eta \right)=\frac{K}{2\cdot {L}_T}\cdot \left(\frac{L_T}{K}-\frac{U_{T1}}{K}-1+F\left({U}_{T1}\right)\cdot \frac{C_2\left({U}_T\right)\cdot \exp \left(\eta \cdot F\left({U}_{T1}\right)\right)+1}{C_2\left({U}_T\right)\cdot \exp \left(\eta \cdot F\left({U}_{T1}\right)\right)-1}\right),\\ {}{p}^1\left({U}_T,\eta \right)=\frac{K}{2\cdot {L}_T}\cdot \left(\frac{L_T}{K}+\frac{U_{T1}}{K}+1-F\left({U}_{T1}\right)\cdot \frac{C_2\left({U}_T\right)\cdot \exp \left(\eta \cdot F\left({U}_{T1}\right)\right)+1}{C_2\left({U}_T\right)\cdot \exp \left(\eta \cdot F\left({U}_{T1}\right)\right)-1}\right),\\ {}\frac{U\left(\eta \right)}{K}=\frac{U_{T1}}{K}-\frac{1}{2}\cdot \left(\frac{L_T}{K}+\frac{U_{T1}}{K}+1-F\left({U}_{T1}\right)\cdot \frac{C_2\left({U}_T\right)\cdot \exp \left(\eta \cdot F\left({U}_{T1}\right)\right)+1}{C_2\left({U}_T\right)\cdot \exp \left(\eta \cdot F\left({U}_{T1}\right)\right)-1}\right),\end{array} $$

where *η* = *t* ⋅ *k*^−^.

The concentration of the *j*th molecular form, *M*_*j*_(*U*_*T*_, *t*), of a protein with *n* independent binding sites in response to a step change in ligand concentration is given by the product of probabilities according to Eq. (9):

41 $$ {M}_j\left({U}_T,t\right)={L}_T\cdot {\displaystyle \prod_{i=0}^{n-1}{p}^{c_i(j)}}\left({U}_T,t\right),\ j=0,\dots, {2}^n-1 $$

where *c*_*i*_(*j*) equals 0 or 1 for free and occupied sites, respectively and $j={\displaystyle \sum_{i=0}^{n-1}{2}^i{c}_i(j)}$.

The kinetics of the amount of ligand bound to a multisite protein with *n* independent binding sites can be written as follows:

42 $$ S\left({U}_T,t\right)={\displaystyle \sum_{j=0}^{2^n-1}{M}_j\left({U}_T,t\right){\displaystyle \sum_{i=0}^{n-1}{c}_i(j)}}. $$

The concentration of free ligand can be written as the difference between the total ligand concentration and the bound ligand concentration (42):

43 $$ U(t)={U}_{T1}-S\left({U}_T,t\right), $$

The probabilities of the *i*th site to be free or occupied respectively for the molecule with *n* independent binding sites in this case:

44 $$ \begin{array}{l}{p}_i^0\left({U}_T,\eta \right)=\frac{K_i}{2\cdot n\cdot {L}_T}\cdot \left(\frac{n\cdot {L}_T}{K_i}-\frac{U_{T1}}{K_i}-1+F\left({U}_{T1}\right)\cdot \frac{C\left({U}_T\right)\cdot \exp \left(\eta \cdot F\left({U}_{T1}\right)\right)+1}{C\left({U}_T\right)\cdot \exp \left(\eta \cdot F\left({U}_{T1}\right)\right)-1}\right),\\ {}{p}_i^1\left({U}_T,\eta \right)=\frac{K_i}{2\cdot n\cdot {L}_T}\cdot \left(\frac{n\cdot {L}_T}{K_i}+\frac{U_{T1}}{K_i}+1-F\left({U}_{T1}\right)\cdot \frac{C\left({U}_T\right)\cdot \exp \left(\eta \cdot F\left({U}_{T1}\right)\right)+1}{C\left({U}_T\right)\cdot \exp \left(\eta \cdot F\left({U}_{T1}\right)\right)-1}\right),\end{array} $$

where $$ F\left({U}_T\right)=\sqrt{{\left(\frac{U_T}{K_i}-\frac{n\cdot {L}_T}{K_i}\right)}^2+2\cdot \left(\frac{U_T}{K_i}+\frac{n\cdot {L}_T}{K_i}\right)+1} $$ and $$ C\left({U}_T\right)=\frac{F\left({U}_{T0}\right)+F\left({U}_{T1}\right)+\frac{U_{T1}}{K_i}-\frac{U_{T0}}{K_i}}{F\left({U}_{T0}\right)-F\left({U}_{T1}\right)+\frac{U_{T1}}{K_i}-\frac{U_{T0}}{K_i}}. $$

The dynamic alterations of the molecular forms with *m* bound out of *n* independent identical binding sites *N*_*m*_(*U*_*T*_, *t*) in response to the total ligand concentration jump from *U*_*T*0_ to *U*_*T*1_ according to Eqs. (19) and (44) are given by:

45 $$ {N}_m\left({U}_T,t\right)={L}_T\cdot {\left({p}^1\left({U}_T,t\right)\right)}^m\cdot {\left({p}^0\left({U}_T,t\right)\right)}^{n-m}. $$

The concentration of multisite protein conformations, *N*_*m*_, bound to *m* ligand molecules as a function of ligand concentration in steady-state is given by:

46 $$ {N}_m\left({U}_T\right)={L}_T{\left(\frac{K}{2n{L}_T}\left(\frac{n{L}_T}{K}+\frac{U_T}{K}+1-F\left({U}_T\right)\right)\right)}^m\cdot {\left(\frac{K}{2n{L}_T}\left(\frac{n{L}_T}{K}-\frac{U_T}{K}-1+F\left({U}_T\right)\right)\right)}^{n-m} $$

The ligand concentrations, *U*_*m*_^*max*^, for the maximal values of intermediate protein conformations, *N*_*m*_(*U*_*m*_^*max*^), can be found by differentiating Eq. (46) with respect to *U*_*T*_ and solving *dN*_*m*_/*dU*_*T*_ = 0 for *U*_*T*_:

47 $$ {U}_m^{max}=m\cdot \left({L}_T+\frac{K}{n-m}\right) $$

The corresponding maximal magnitudes for the intermediate conformations are given by:

48 $$ {N}_m\left({U}_m^{max}\right)={L}_T\cdot \frac{m^m}{n^n}\cdot {\left(n-m\right)}^{n-m} $$

The half maximal effective ligand concentration, *U*_0_^0.5^ and *U*_*n*_^0.5^, when the protein species equal half of the total concentration *L*_*T*_, for the apo- and saturated multisite protein conformations respectively, in this case are given by:

49 $$ \begin{array}{l}{U}_0^{0.5}=\left(1-\sqrt[n]{0.5}\right)\cdot \left(n\cdot {L}_T+\frac{K}{\sqrt[n]{0.5}}\right),\\ {}{U}_n^{0.5}=\sqrt[n]{0.5}\cdot \left(n\cdot {L}_T+\frac{K}{1-\sqrt[n]{0.5}}\right).\end{array} $$

Differentiating Eq. (45) with respect to *t* and solving for *dN*_*m*_/*dt* = 0 yields the time *τ*_*m*_^*max*^ when the concentration of multisite protein conformations, *N*_*m*_, bound to *m* ligand molecules is maximal:

50 $$ {\tau}_m^{max}=H\left(V(m)\right), $$

where $$ H(x)=\frac{ \ln \left(\frac{1+\frac{U_{T1}}{K}+F\left({U}_{T1}\right)+x}{C\left({U}_T\right)\cdot \left(1+\frac{U_{T1}}{K}-F\left({U}_{T1}\right)+x\right)}\right)}{F\left({U}_{T1}\right)\cdot {k}^{-}} $$ and $$ V(m)=\frac{L_T}{K}\cdot \left(n-2m\right). $$

Equation (50) has an asymptote $\frac{U_{T1}}{K}$ for $1+\frac{U_{T1}}{K}-F\left({U}_{T1}\right)+x=0$:

51 $$ {q}_m=m\cdot \left(\frac{L_T}{K}+\frac{1}{n-m}\right), $$

Equation (45) is further used to define the time, *τ*_0_^0.5^, required for the apo- form, *N*_0_, to reach half of the growth concentration and the time period, *τ*_*n*_^0.5^, required for the fully saturated protein species to gain half of the growth concentration:

52 $$ \begin{array}{l}{\tau}_0^{0.5}=H(W),\\ {}{\tau}_n^{0.5}=H(Y),\end{array} $$

where

$$ \begin{array}{l}W=\sqrt[n]{\frac{{\left(-\frac{U_{T1}}{K}+\frac{L_T}{K}\cdot n+F\left({U}_{T1}\right)-1\right)}^n+{\left(-\frac{U_{T0}}{K}+\frac{L_T}{K}\cdot n+F\left({U}_{T0}\right)-1\right)}^n}{2}}-\frac{L_T}{K}\cdot n,\\ {}Y=\frac{L_T}{K}\cdot n-\sqrt[n]{\frac{{\left(\frac{U_{T1}}{K}+\frac{L_T}{K}\cdot n-F\left({U}_{T1}\right)+1\right)}^n+{\left(\frac{U_{T0}}{K}+\frac{L_T}{K}\cdot n-F\left({U}_{T0}\right)+1\right)}^n}{2}}.\end{array} $$

### Ethics (and consent to participate)

Not applicable.

### Consent to publish

Not applicable.

### Availability of data and materials

The data supporting the findings of this work are contained within the manuscript.

## Abbreviations

Ca^2+^: calcium ion
CaM: calmodulin
CaMKII: calcium/calmodulin-dependent protein kinase II
CaN: calcineurin
CR: calretenin
PP2B: protein phosphatase 2B, or calcineurin
TnC: troponin C

## References

[1] Maki M, Maemoto Y, Osako Y, Shibata H (2012). Evolutionary and physical linkage between calpains and penta-EF-hand Ca2 + -binding proteins. FEBS J.

[2] Chillakuri CR, Sheppard D, Lea SM, Handford PA (2012). Notch receptor-ligand binding and activation: insights from molecular studies. Semin Cell Dev Biol.

[3] Grabarek Z (2011). Insights into modulation of calcium signaling by magnesium in calmodulin, troponin C and related EF-hand proteins. Biochim Biophys Acta.

[4] Yap KL, Ames JB, Swindells MB, Ikura M (1999). Diversity of conformational states and changes within the EF-hand protein superfamily. Proteins Struct Funct Genet.

[5] Kawasaki H, Nakayama S, Kretsinger RH (1998). Classification and evolution of EF-hand proteins. Biometals.

[6] Canepari M, Maffei M, Longa E, Geeves M, Bottinelli R (2012). Actomyosin kinetics of pure fast and slow rat myosin isoforms studied by in vitro motility assay approach. Exp Physiol.

[7] Oz S, Benmocha A, Sasson Y, Sachyani D, Almagor L (2013). Competitive and Non-competitive Regulation of Calcium-dependent Inactivation in Ca(V)1.2 L-type Ca2+ Channels by Calmodulin and Ca2 + -binding Protein 1. J Biol Chem.

[8] Wei XY, Pan S, Lang WH, Kim HY, Schneider T (1995). Molecular Determinants of Cardiac Ca2+ Channel Pharmacology - Subunit Requirement for the High-Affinity and Allosteric Regulation of Dihydropyridine Binding. J Biol Chem.

[9] McCarron JG, Chalmers S, Olson ML, Girkin JM (2012). Subplasma Membrane Ca2+ Signals. Iubmb Life.

[10] Kotov NV, Bates DG, Gizatullina AN, Gilaziev B, Khairullin RN (2011). Computational modelling elucidates the mechanism of ciliary regulation in health and disease. BMC Syst Biol.

[11] Fuchs F, Grabarek Z (2011). The Ca/Mg Sites of Troponin C Can Modulate Crossbridge-Mediated Thin Filament Activation in Rat Cardiac Myofibrils. Biophys J.

[12] Pearson DS, Swartz DR, Geeves MA (2008). Fast pressure jumps can perturb calcium and magnesium binding to troponin C F29W. Biochemistry.

[13] Shifman JM, Choi MH, Mihalas S, Mayo SL, Kennedy MB (2006). Ca2+/calmodulin-dependent protein kinase II (CaMKII) is activated by calmodulin with two bound calciums. Proc Natl Acad Sci U S A.

[14] Guo Q, Shen Y, Lee YS, Gibbs CS, Mrksich M (2005). Structural basis for the interaction of Bordetella pertussis adenylyl cyclase toxin with calmodulin. EMBO J.

[15] Robison J C, RJ. Calcium/Calmodulin-Dependent Protein Kinases. In: WJ Lennarz ML, editor. Encyclopedia of Biological Chemistry: Elsevier Inc. Cambridge, Massachusetts: Academic Press; 2004. pp. 281-286.

[16] Bhattacharya S, Bunick CG, Chazin WJ (2004). Target selectivity in EF-hand calcium binding proteins. Biochim Biophys Acta.

[17] Nelson MR, Thulin E, Fagan PA, Forsen S, Chazin WJ (2002). The EF-hand domain: a globally cooperative structural unit. Protein Sci.

[18] Hoeflich KP, Ikura M (2002). Calmodulin in action: diversity in target recognition and activation mechanisms. Cell.

[19] Schumacher MA, Rivard AF, Bachinger HP, Adelman JP (2001). Structure of the gating domain of a Ca2 + -activated K+ channel complexed with Ca2+/calmodulin. Nature.

[20] Rodney GG, Moore CP, Williams BY, Zhang JZ, Krol J (2001). Calcium binding to calmodulin leads to an N-terminal shift in its binding site on the ryanodine Receptor. J Biol Chem.

[21] Yap KL, Ames JB, Swindells MB, Ikura M (1999). Diversity of conformational states and changes within the EF-hand protein superfamily. Proteins.

[22] Valeyev NV, Bates DG, Heslop-Harrison P, Postlethwaite I, Kotov NV (2008). Elucidating the mechanisms of cooperative calcium-calmodulin interactions: a structural systems biology approach. BMC Syst Biol.

[23] Valeyev NV, Heslop-Harrison P, Postlethwaite I, Gizatullina AN, Kotov NV (2009). Crosstalk between G-protein and Ca2+ pathways switches intracellular cAMP levels. Mol Biosyst.

[24] Valeyev NV, Heslop-Harrison P, Postlethwaite I, Kotov NV, Bates DG (2008). Multiple calcium binding sites make calmodulin multifunctional. Mol Biosyst.

[25] Hyde JR, Kezunovic N, Urbano FJ, Garcia-Rill E. Spatiotemporal properties of high speed calcium oscillations in the pedunculopontine nucleus. J Appl Physiol. 2013;115:1402-1414. 10.1152/japplphysiol.00762.2013 PMC3841830 23990242

[26] Bading H (2013). Nuclear calcium signalling in the regulation of brain function. Nat Rev Neurosci.

[27] Semenov I, Xiao S, Pakhomova ON, Pakhomov AG (2013). Recruitment of the intracellular Ca(2+) by ultrashort electric stimuli: The impact of pulse duration. Cell Calcium.

[28] Faas GC, Schwaller B, Vergara JL, Mody I (2007). Resolving the fast kinetics of cooperative binding: Ca2+ buffering by calretinin. PLoS Biol.

[29] Adair GS (1925). The hemoglobin system. VI. The oxygen dissociation curve of hemoglobin. J Biol Chem.

[30] Klotz IM, Hunston DL (1975). Protein interactions with small molecules. Relationships between stoichiometric binding constants, site binding constants, and empirical binding parameters. J Biol Chem.

[31] Faas GC, Raghavachari S, Lisman JE, Mody I (2011). Calmodulin as a direct detector of Ca2+ signals. Nat Neurosci.

[32] Andre I, Kesvatera T, Jonsson B, Akerfeldt KS, Linse S (2004). The role of electrostatic interactions in calmodulin-peptide complex formation. Biophys J.

[33] Andre I, Kesvatera T, Jonsson B, Linse S (2006). Salt enhances calmodulin-target interaction. Biophys J.

[34] Zhang M, Tanaka T, Ikura M (1995). Calcium-induced conformational transition revealed by the solution structure of apo calmodulin. Nat Struct Biol.

[35] Chattopadhyaya R, Meador WE, Means AR, Quiocho FA (1992). Calmodulin structure refined at 1.7 A resolution. J Mol Biol.

[36] Aloy P, Russell RB (2006). Structural systems biology: modelling protein interactions. Nat Rev Mol Cell Biol.

[37] Vetter SW, Leclerc E (2003). Novel aspects of calmodulin target recognition and activation. Eur J Biochem.

[38] Nelson MR, Chazin WJ (1998). An interaction-based analysis of calcium-induced conformational changes in Ca2+ sensor proteins. Protein Sci.

[39] Hill AV (1910). The possible effects of the aggregation of the molecules of hemoglobin on its dissociation curves. J Physiol.

[40] Weiss JN (1997). The Hill equation revisited: uses and misuses. FASEB J.

[41] Yang W, Lee HW, Hellinga H, Yang JJ (2002). Structural analysis, identification, and design of calcium-binding sites in proteins. Proteins.

[42] Friedberg F (1988). Calcium Binding Protein Families: The ‘E-F Hand’ Family. Biochem Educ.

[43] Kissinger CR, Parge HE, Knighton DR, Lewis CT, Pelletier LA (1995). Crystal-Structures of Human Calcineurin and the Human Fkbp12-Fk506-Calcineurin Complex. Nature.

[44] Kilhoffer MC, Roberts DM, Adibi AO, Watterson DM, Haiech J (1988). Investigation of the mechanism of calcium binding to calmodulin. Use of an isofunctional mutant with a tryptophan introduced by site-directed mutagenesis. J Biol Chem.

[45] Holzer H (1981). Metabolic Interconversion of Enzymes 1980 International Titisee Conference October 1st - 5th, 1980. Proceedings in Life Sciences, 0172-6625.

[46] Klee CB, Crouch TH, Krinks MH (1979). Calcineurin: a calcium- and calmodulin-binding protein of the nervous system. Proc Natl Acad Sci U S A.

[47] Crouch TH, Klee CB (1980). Positive cooperative binding of calcium to bovine brain calmodulin. Biochemistry.

[48] Klabunde RE (2012). Cardiovascular physiology concepts.

[49] Stein WD, Lieb WR (1986). Transport and diffusion across cell membranes.

[50] Ryerson S, Enciso GA (2014). Ultrasensitivity in independent multisite systems. J Math Biol.

[51] Bazanovas AN, Evstifeev AI, Khaiboullina SF, Sadreev II, Skorinkin AI (2015). Erythrocyte: A systems model of the control of aggregation and deformability. Biosystems.

[52] Bode W, Schwager P (1975). The single calcium-binding site of crystallin bovin beta-trypsin. FEBS Lett.

[53] Minowa O, Yagi K (1984). Calcium binding to tryptic fragments of calmodulin. J Biochem.

[54] Linse S, Helmersson A, Forsen S (1991). Calcium binding to calmodulin and its globular domains. J Biol Chem.

[55] Cimmperman P, Baranauskiene L, Jachimoviciute S, Jachno J, Torresan J (2008). A quantitative model of thermal stabilization and destabilization of proteins by ligands. Biophys J.

[56] Almagor H, Levitzki A (1990). Analytical determination of receptor-ligand dissociation constants of two populations of receptors from displacement curves. Proc Natl Acad Sci U S A.

[57] Kragh-Hansen U (1983). Graphical analysis of competitive binding of comparable concentrations of ligand, inhibitor and protein. Ligand binding to serum albumin. Biochem Pharmacol.

[58] Heeley DH, Belknap B, White HD (2006). Maximal activation of skeletal muscle thin filaments requires both rigor myosin S1 and calcium. J Biol Chem.

[59] Stefan MI, Edelstein SJ, Le Novere N (2008). An allosteric model of calmodulin explains differential activation of PP2B and CaMKII. Proc Natl Acad Sci U S A.

[60] Nishiyama M, Kimura Y, Nishiyama Y, Terazima M (2009). Pressure-induced changes in the structure and function of the kinesin-microtubule complex. Biophys J.

[61] Pearson DS, Holtermann G, Ellison P, Cremo C, Geeves MA (2002). A novel pressure-jump apparatus for the microvolume analysis of protein-ligand and protein-protein interactions: its application to nucleotide binding to skeletal-muscle and smooth-muscle myosin subfragment-1. Biochem J.

[62] Zhu T, Beckingham K, Ikebe M (1998). High affinity Ca2+ binding sites of calmodulin are critical for the regulation of myosin Ibeta motor function. J Biol Chem.

[63] Rusnak F, Mertz P (2000). Calcineurin: form and function. Physiol Rev.

[64] Lu HE, MacGillavry HD, Frost NA, Blanpied TA (2014). Multiple spatial and kinetic subpopulations of CaMKII in spines and dendrites as resolved by single-molecule tracking PALM. J Neurosci.

[65] Lee SJ, Escobedo-Lozoya Y, Szatmari EM, Yasuda R (2009). Activation of CaMKII in single dendritic spines during long-term potentiation. Nature.

[66] Shaikh TR, Barnard D, Meng X, Wagenknecht T (2009). Implementation of a flash-photolysis system for time-resolved cryo-electron microscopy. J Struct Biol.

[67] Frieden C, Hoeltzli SD, Ropson IJ (1993). NMR and protein folding: equilibrium and stopped-flow studies. Protein Sci.

[68] Nagerl UV, Novo D, Mody I, Vergara JL (2000). Binding kinetics of calbindin-D(28 k) determined by flash photolysis of caged Ca(2+). Biophys J.

[69] Plaza-Menacho I, Barnouin K, Goodman K, Martinez-Torres RJ, Borg A (2014). Oncogenic RET kinase domain mutations perturb the autophosphorylation trajectory by enhancing substrate presentation in trans. Mol Cell.

[70] Sadreev II, Chen MZ, Welsh GI, Umezawa Y, Kotov NV (2014). A systems model of phosphorylation for inflammatory signaling events. PLoS One.

[71] Cutillas PR, Geering B, Waterfield MD, Vanhaesebroeck B (2005). Quantification of gel-separated proteins and their phosphorylation sites by LC-MS using unlabeled internal standards: analysis of phosphoprotein dynamics in a B cell lymphoma cell line. Mol Cell Proteomics.

[72] Zorba A, Buosi V, Kutter S, Kern N, Pontiggia F (2014). Molecular mechanism of Aurora A kinase autophosphorylation and its allosteric activation by TPX2. Elife.

